# Physiological and Multi-Omics Analysis in Leaves of *Solanum americanum* in Response to Cd Toxicity

**DOI:** 10.3390/plants14142131

**Published:** 2025-07-10

**Authors:** Jiao Zhou, Jun-Gang Zhu, Peng Xiao, Kai-Lu Wang, Qian Xu, Meng-Xi Wu, Yuan-Zhi Pan

**Affiliations:** 1 College of Landscape Architecture, Sichuan Agricultural University, Chengdu 611130, China; tracyzhou66@outlook.com (J.Z.); sunnyglow2024@outlook.com (P.X.);; 2 Department of Landscape Architecture, School of Architecture and Planning, Foshan University, Foshan 528000, China; 3 College of Forestry, Sichuan Agricultural University, Chengdu 611130, China

**Keywords:** transcriptomic, proteomic, metabolomics, Cd stress, leaf, *Solanum americanum*

## Abstract

Phytoremediation is a green economic method to address soil cadmium (Cd) pollution, and *Solanum americanum* is considered a potential phytoremediation candidate. However, the underlying Cd response mechanisms of *S. americanum* remain unclear. In the current study, a hydroponic experiment with 160 μmol/L Cd stress was conducted, physiological and molecular indices were measured to explore the response of *S. americanum* leaves to Cd stress at different time points (0, 3, and 7 days). Our findings revealed that Cd stress inhibited plant growth. Moreover, Cd stress significantly increased Cd accumulation, as well as Chla content, Chla/b, activities of SOD and POD, and elevated MDA content in the leaves. Furthermore, transcriptomics, proteomics, and metabolomics analyses revealed 17,413 differentially expressed genes (DEGs), 1421 differentially expressed proteins (DEPs), and 229 differentially expressed metabolites (DEMs). Meanwhile, integrative analyses of multi-omics data revealed key proteins involved in response to Cd stress, including POD, PAL, F5H, COMT, and CAD for phenylpropanoid biosynthesis, as well as GAPA, FBP, and FBA for photosynthesis pathways. Additionally, conjoint analyses highlighted that upregulated phenylpropanoid metabolism and photosynthesis alleviated Cd toxicity, playing vital roles in enhancing Cd tolerance in leaves. A conceptual molecular regulatory network of leaves in the response to Cd toxicity was proposed. This comprehensive study will provide detailed molecular-scale insights into the Cd response mechanisms in *S. americanum*.

## 1. Introduction

Cd, a non-essential element for plants, is characterized by its high mobility, marked solubility, and severe toxicity [1,2]. Due to its similarity to essential elements such as zinc (Zn), calcium (Ca), magnesium (Mg), and iron (Fe) [3], Cd can inhibit plant growth by competing for ion channels or transporter proteins of these elements [4]. Human or animal health will be directly threatened when the food chain is contaminated by Cd [5,6,7]. Therefore, it is an urgent task to address soil Cd contamination. As a green economic method for polluted soil remediation, phytoremediation (using hyperaccumulators or hyper-tolerant plants), holds significant practical importance in addressing heavy metal (HM) soil contamination [8,9]. The molecular mechanisms of absorption, transformation, and fixation of HMs by plants are crucial for phytoremediation purposes [10]. Studying Cd-related genes, proteins, and metabolites in plants can provide molecular resources for phytoremediation [11], which will certainly lay a theoretical foundation for soil remediation.

Usually, even trace levels of Cd can disrupt the metabolic balance of plants by causing reactive oxygen species (ROS) toxicity [12,13,14,15]. Previous studies have shown that Cd generally has a direct or indirect negative impact on the physiological and molecular processes of plants, including tissue growth, nutrient absorption, photosynthesis, element balance, antioxidant enzyme activity, ROS accumulation, biomass reduction, and molecular pathway perturbation [10,13,16,17,18,19,20,21]. Interestingly, the difference between healthy plants and Cd hyperaccumulators is that the latter have evolved considerable detoxification and tolerance mechanisms to defend against Cd stress and maintain normal physiological functions, and including cell wall binding, vacuolar sequestration, ROS scavenging, and ion chelation [18,22,23,24,25]. Among these, cell wall binding, as a significant physical strategy for effectively restraining Cd entry into plant cells, has been previously demonstrated [22]. Lignin is the principal structural component of plant cell walls and is synthesized through the phenylpropanoid pathway [26]. Recent studies have highlighted the importance of phenylpropanoid biosynthesis in mitigating Cd toxicity [19,27]. Furthermore, leaves are the most efficient organs for photosynthesis in plants [28,29]. Photosynthesis provides the energy foundation for the biosphere by converting CO_2_ into organic compounds and supports plant growth [30]. Cd has detrimental effects on the photosynthetic membrane, electron transport chain, and Calvin cycle for leaves [31]. However, studies on certain Cd/Zn hyperaccumulators have demonstrated that many steps in their photosynthesis process are Cd-tolerant, while relevant DEGs or DEPs are about transcription and translation, electron transport and ATP synthesis. Photosystem II and photosystem I respond to Cd stress by upregulating their expression level [32,33,34,35]. Previous studies on Cd detoxification mechanisms have focused on specific plants. Therefore, further investigation is needed to determine whether these mechanisms exist in different candidate species for phytoremediation.

*Solanum americanum* Mill. (formerly known as *Solanum photeinocarpum* L.) has been identified as a potential Cd hyperaccumulator [36]; moreover, it is also widely consumed as a fruit, local leafy vegetable, and medicinal plant in some regions [37]. Recent studies have investigated the physio-biochemical mechanisms of *S. americanum* in response to Cd, including Cd accumulation, biomass, photosynthesis, ROS, and antioxidant defense [36,38,39,40]. Our previous study found that the Chla/b and initial fluorescence (Fo) of *S. americanum* seedlings were increased with increasing Cd stress [41]. However, the underlying molecular mechanisms of *S. americanum* response to Cd stress remain elusive, especially in leaves. In recent decades, techniques such as RNA-seq, Label-free, and LC-MS have been widely applied to study the response mechanisms to Cd stress in different plants, including rice [19], *Sedum plumbizincicola* [42], *Abelmoschus manihot* [43], and *Solanum nigrum* [22]. Therefore, utilizing these omics technologies can help elucidate Cd-response mechanisms of *S. americanum*. In this study, an integrated analysis of physiological, transcriptomic, proteomic, and metabolomic processes was performed to gain insights into the short-term acclimation of *S. americanum* to Cd stress. The objectives of this study are (1) to reveal the changes in physiological, transcriptomic, proteomic, and metabolomic processes in leaves of *S. americanum* in response to Cd stress, and (2) to explore a potential regulatory network between genes, proteins, and metabolites in *S. americanum* under Cd stress. These results will provide a better understanding of Cd detoxification mechanisms in *S. americanum* and further identify important responsive candidate genes for phytoremediation.

## 2. Results

### 2.1. Physiological Effects in Leaf Under Cd Stress

**Figure 1:**
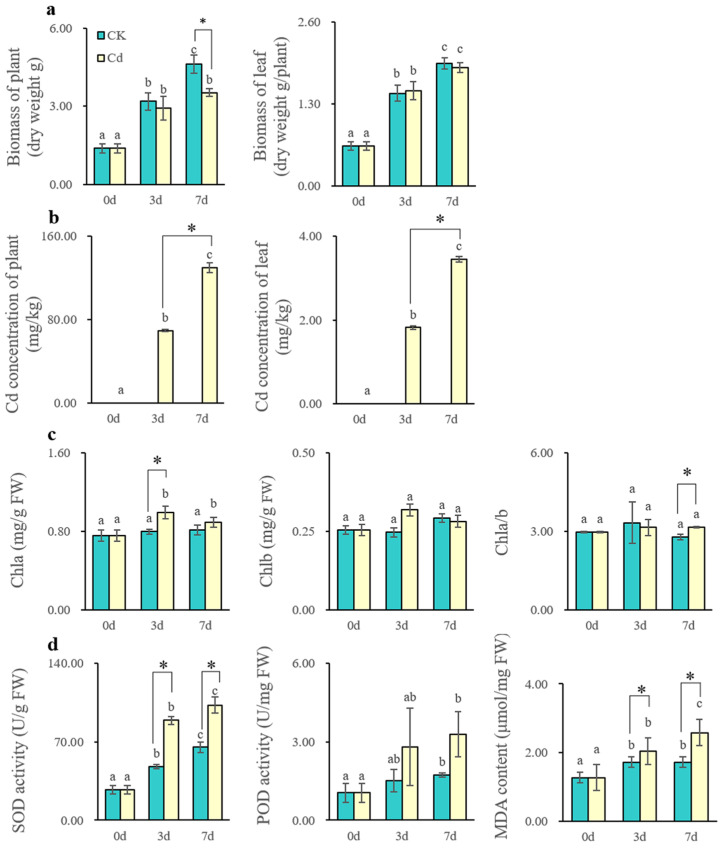
Physiological response in leaves of *S. americanum* under Cd stress. (**a**) Biomass of plant and leaf; (**b**) Cd concentration of plant and leaf; (**c**) content of Chla, Chlb, and Chla/b in leaf; (**d**) activity of SOD, POD, and MDA content in leaf; values are the mean ± SE (n = 3), different lowercase letters indicate significant differences at different time points in the same tissue (*p* < 0.05), and “*” indicates significant difference between Cd-treated and control plants.

After 160 μM Cd treatment, the biomass of Cd-treated *S. americanum* significantly decreased by 23.54% at 7 d compared to the control (*p* < 0.05), biomass of leaves decreased by 10.34% at 7 d (Figure 1a). In addition, the Cd content in plants and leaves significantly increased (*p* < 0.05); Cd content in both plants and leaves was approximately doubled from 3 d to 7 d, with the average Cd content in plants reaching 129.67 mg/kg at 7 d, which exceeds the known upper tolerance threshold of 100 mg/kg of Cd hyperaccumulators (Figure 1b). Furthermore, Cd stress led to an increase in Chla content in leaves by 9.42–24.39%. Chlb content increased at 3 d and then decreased at 7 d. Chla/b increased by 13.40% at 7 d (Figure 1c). Additionally, SOD significantly increased by 57.62–86.76% (*p* < 0.05), POD increased by 85.05–89.21%. Moreover, the MDA content significantly increased by 18.68–49.77% (*p* < 0.05) (Figure 1d).

Furthermore, the content of Na, Mg, and Fe increased after Cd treatment (Figure S1a,d,h), while the content of K, Mn, and Cu decreased (Figure S1b,e,g). The content of Zn and Ca did not change significantly (*p* > 0.05) (Figure S1c,f). In conclusion, Cd stress influenced the distribution of K, Na, Mg, Mn, Cu, and Fe in our experiment.

### 2.2. The Response of Leaf Transcriptomes to Cd Toxicity

An average of 23.03 million clean reads were obtained from leaf samples, the GC, Q20 and Q30 values of all clean reads were greater than 42%, 97%, and 93%, respectively (Table S2). This indicated the high reliability of the sequencing results. De novo assembly of clean reads generated a reference transcriptome of 567,870 unigenes. The mean length, N50, and N90 of unigenes were 370 bp, 314 bp, and 231 bp, respectively (Table S3). All the unigenes were successfully annotated in seven public databases (Table S4). Furthermore, the expression trends of selected DEGs, as determined by qPCR analysis, were similar to those obtained from Illumina sequencing (Figure S2), indicating the reliability of the RNA-seq data.

**Figure 2:**
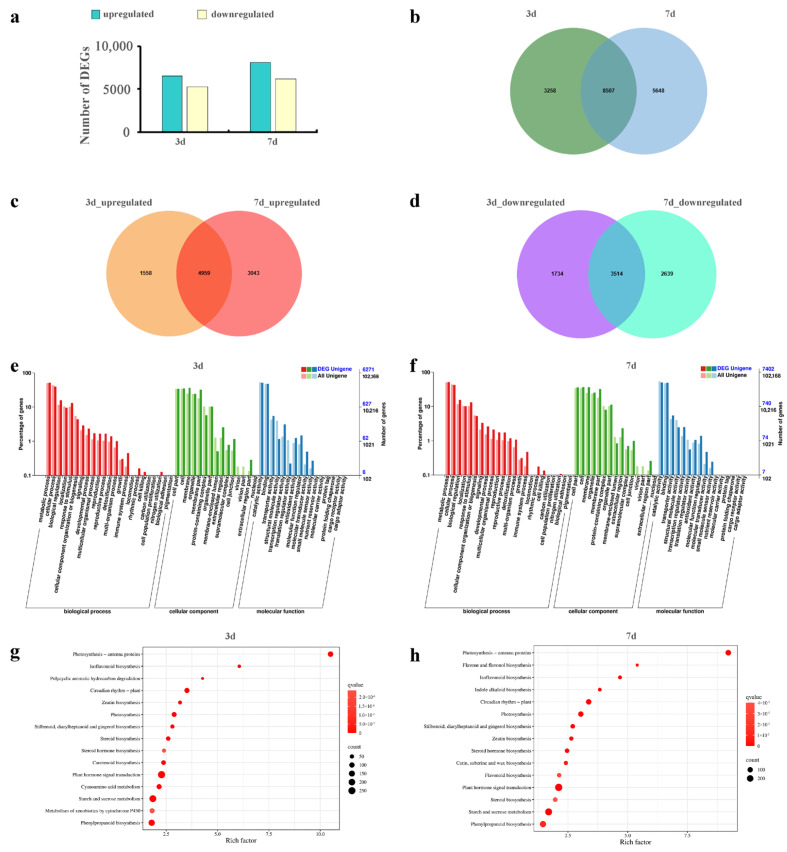
Analysis of gene expression levels in the leaves of *S. americanum* under Cd stress. (**a**) The number of Cd-responsive DEGs in leaves at different Cd stress time points; (**b**) Venn diagram summarizing all identified DEGs; Venn diagram summarizing overlapping upregulated (**c**) and downregulated (**d**) DEGs in leaves at different Cd stress time points; (**e**,**f**) histograms showing GO enrichment of DEGs in leaves at different Cd stress time points; (**g**,**h**) bubble diagrams showing KEGG pathway annotation of DEGs in leaves at different Cd stress time points (showing the top 15 most significantly enriched pathways, *p* < 0.05).

A total of 17,413 DEGs were identified. Additionally, comparative analysis of DEGs revealed that a total of 11,765 (6517 upregulated and 5248 downregulated) and 14,155 (8002 upregulated and 6153 downregulated) DEGs at 3 d and 7 d (Figure 2a,b), respectively. Among them, 4959 upregulated (Figure 2c) and 3514 downregulated (Figure 2d) DEGs were screened as commonly expressed, indicating that the leaves of *S. americanum* regulated the expression of numerous genes to cope with Cd stress.

The GO enrichment analysis was carried out to find the specific biological functions of DEGs, all the annotated DEGs with enriched GO terms were mainly involved in “metabolic process”, “cellular process”, and “biological regulation” (BP), “cell part”, “cell”, and “membrane” (CC), “catalytic activity”, “binding”, and “transporter activity” (MF) (Figure 2e,f). GO analysis indicated that the leaves of *S. americanum* affect the defense mechanism and organization of cell part to respond to Cd stress.

To elucidate the biological effects of Cd stress, all the DEGs were mapped to the KEGG database for enrichment analysis. There were 11 pathways commonly enriched in the top 15 significantly enriched KEGG pathway (*p* < 0.05), including “photosynthesis-antenna proteins and Photosynthesis”, “phenylpropanoid biosynthesis”, “isoflavonoid biosynthesis”, “stilbenoid, diarylheptanoid and gingerol biosynthesis”, “starch and sucrose metabolism”, “zeatin biosynthesis”, “plant hormone signal transduction”, “steroid biosynthesis”, and “steroid hormone biosynthesis” (Figure 2g,h). Which indicated that *S. americanum* regulated photosynthesis metabolism, carbohydrate metabolism, signal transduction and lipid metabolism to reduce Cd toxicity on leaves.

### 2.3. The Response of Leaf Proteomes to Cd Toxicity

**Figure 3:**
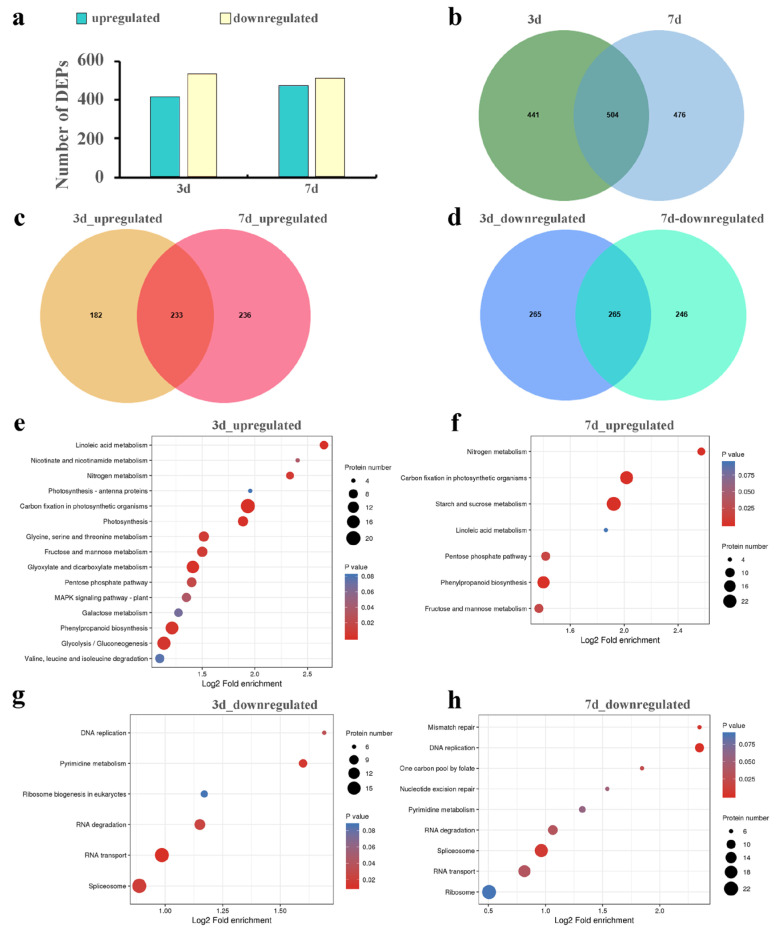
Analysis of protein expression levels in the leaves of *S. americanum* under Cd stress. (**a**) The number of Cd-responsive DEPs in leaves at different Cd stress time points; (**b**) Venn diagram summarizing all the identified DEPs; Venn diagram summarizing overlapping upregulated (**c**) and downregulated (**d**) DEPs in leaves at different Cd stress time points; (**e**,**f**) KEGG enrichment analysis of upregulated DEPs in leaves at different Cd stress time points; (**g**,**h**) KEGG enrichment of downregulated DEPs in leaves at different Cd stress time points.

It was identified that a total of 9029 quantifiable proteins (Table S5), including 1421 DEPs (Figure 3). The comparative analysis of DEPs revealed 945 (415 upregulated and 530 downregulated) and 980 (469 upregulated and 511 downregulated) DEPs for 3 d and 7 d (Figure 3a,b), respectively. Of these, 233 upregulated (Figure 3c) and 265 downregulated (Figure 3d) DEPs were commonly expressed. Additionally, the expression trends of selected DEPs in PRM were consistent with those observed in quantitative proteomics (Figure S3), confirming the reliability of the Label-free data.

The functions of DEPs were classified according to GO classifications. The upregulated DEPs were predominantly associated with phosphatase activity of “fructose 1,6-bisphosphate 1”, “sugar”, and “carbohydrate “ in MF (Figure S4a,b); “chloroplast”, “plastid”, and “chloroplast part” in CC (Figure S4c,d); and “glucose metabolic process”, “gluconeogenesis”, and “hexose biosynthetic process” in BP (Figure S4e,f). Subsequently, KEGG enrichment analysis was also performed, and the upregulated DEPs were significantly (*p* < 0.05) enriched in “carbon fixation in photosynthetic organisms”, “phenylpropanoid biosynthesis”, “nitrogen metabolism”, “fructose and mannose metabolism”, and “pentose phosphate pathway” (Figure 3e,f). These results suggest that Cd stress affects energy metabolism, carbohydrate metabolism, antioxidant activity, and biosynthesis of other secondary metabolites in the leaves of *S. americanum*. It may enhance pathways such as carbon and nitrogen metabolism, photosynthesis, and phenylpropanoid biosynthesis, thereby improving Cd tolerance.

In addition, for the downregulated DEPs, GO analysis indicates they were predominantly associated with “RNA binding” in MF (Figure S5a,b), “nuclear part” in CC (Figure S5c,d), and “RNA splicing” in BP (Figure S5e,f). KEGG enrichment analysis suggests that downregulated DEPs were significantly (*p* < 0.05) enriched in “RNA transport”, “spliceosome”, “RNA degradation”, and “DNA replication”. These results indicate that Cd stress affects the biogenesis of *S. americanum*, which may reduce specific processes in leaves to cope with Cd stress.

### 2.4. The Response of Leaf Metabolomes to Cd Toxicity

**Figure 4:**
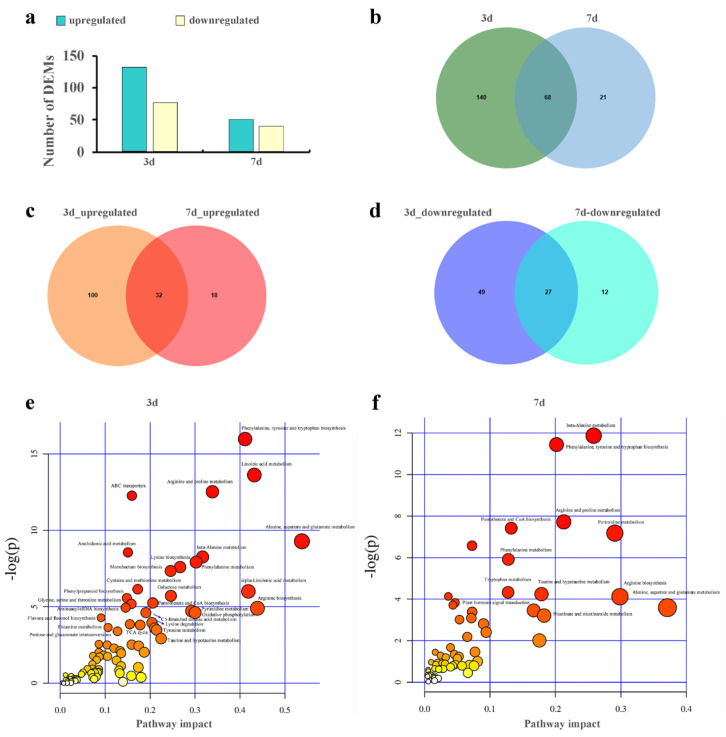
Analysis of metabolite abundance levels in leaves of *S. americanum* under Cd stress. (**a**) The number of Cd-responsive DEMs in leaves at different Cd stress time points; (**b**) Venn diagram summarizing all the identified DEMs; Venn diagram summarizing the overlapping upregulated (**c**) and downregulated (**d**) DEMs in leaves at different Cd stress time points; (**e**,**f**) bubble diagram showing KEGG pathway annotations of DEMs in leaves at different Cd stress time points.

To investigate the influence of Cd accumulation on metabolic activity in the leaves of *S. americanum*, multivariate PCA (Figure S6a,b,e,f) and OPLS-DA (Figure S6c,d,g,h) were subsequently applied. PCA results showed that biological replicates from each group clustered together, demonstrating the stability and reliability of the sequencing results and confirming good reproducibility of samples in both positive and negative ion modes (Figure S6a,b,e,f). It was identified that a total of 229 DEMs using LC-MS methods (Figure 4a,b). As the duration of Cd exposure increased, distinct differences emerged among leaf samples. A total of 208 (132 upregulated and 76 downregulated) and 89 (50 upregulated and 39 downregulated) DEMs were identified at 3 d and 7 d, respectively (Figure 4c,d). These DEMs were mainly classified into lipid (24.02%), organic acid (23.58%), organic oxygen compounds (12.66%), organic heterocyclic compounds (10.92%), phenylpropanoids and polyketides (6.99%) (Table S6). KEGG enrichment analysis of metabolite abundance levels was conducted to confirm the important metabolic pathways related to the responses of *S. americanum* leaves to Cd stress (Figure 4e,f): 60 pathways were commonly identified, mainly classified as amino acid metabolism (30%), biosynthesis of other secondary metabolites (15%), carbohydrate metabolism (15%), energy metabolism (8.33%), lipid metabolism (8.33%), membrane transport (1.67%), and signal transduction (1.67%).

In addition, comprehensive analysis results revealed that nine pathways were significantly perturbed (impact value > 0.1 and raw *p* < 0.05) (Table S7), including seven amino acid pathways: “arginine biosynthesis”, “alanine, aspartate and glutamate metabolism”, “arginine and proline metabolism”, “phenylalanine, tyrosine and tryptophan biosynthesis”, “phenylalanine metabolism”, “β-alanine metabolism”, and “taurine and hypotaurine metabolism”. These results indicate that amino metabolites played a crucial role in the response of *S. americanum* leaves to Cd stress.

### 2.5. The Leaf Regulatory Network in Response to Cd Toxicity

**Figure 5:**
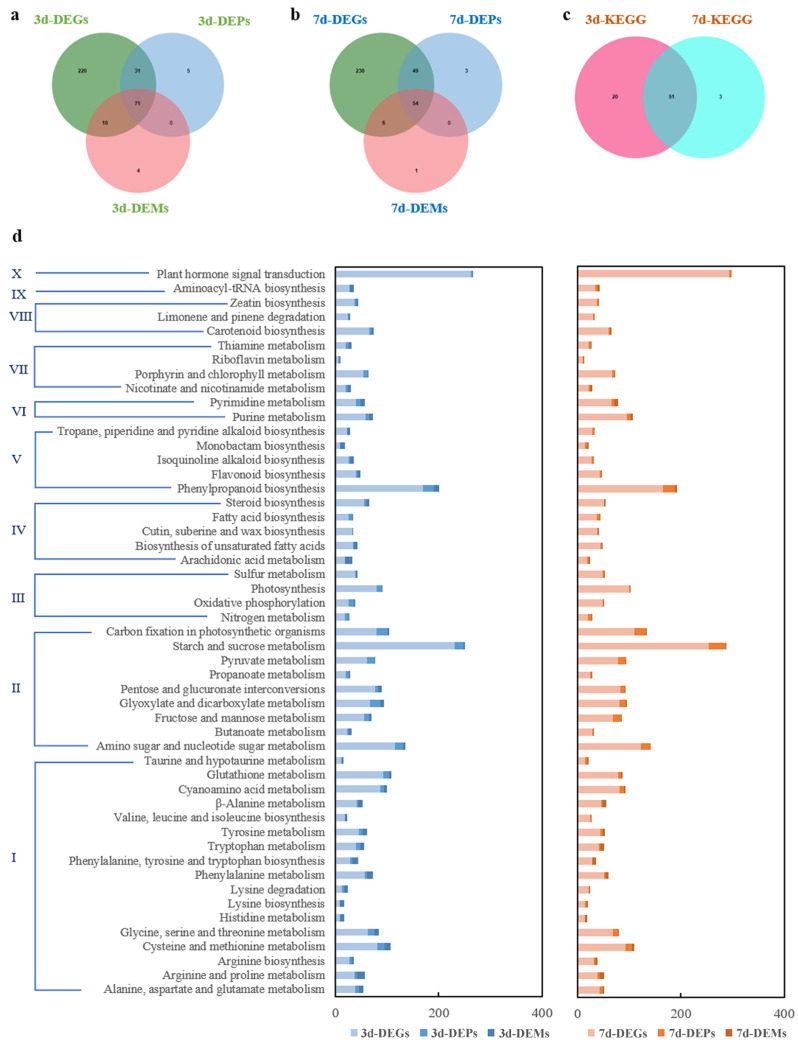
Joint analysis of transcriptomic, proteomic, and metabolomic data in leaves of *S. americanum* under Cd stress. (**a**) Venn diagram of KEGG pathways in DEGs/DEPs/DEMs for 3 d; (**b**) Venn diagram of KEGG pathway in DEGs/DEPs/DEMs for 7 d; (**c**) Venn diagram of KEGG pathway for 3 d and 7 d; (**d**) Common KEGG pathways annotated by Cd-responsive DEGs/DEPs/DEMs in leaves. Pathway classification: Ⅰ. Amino acid metabolism; Ⅱ. Carbohydrate metabolism; Ⅲ. Energy metabolism; Ⅳ. Lipid metabolism; Ⅴ. Biosynthesis of other secondary metabolites; Ⅵ. Nucleotide metabolism; Ⅶ. Metabolism of cofactors and vitamins; Ⅷ. Metabolism of terpenoids and polyketides; Ⅸ. Translation, and Ⅹ. Signal transduction.

Results of the joint analysis based on Cd-responsive DEGs, DEPs, and DEMs indicated that a total of 51 pathways were annotated (Figure 5a–c), which include 10 categories (Figure 5d), such as amino acid metabolism (17), carbohydrate metabolism (9), energy metabolism (4), lipid metabolism (5), biosynthesis of other secondary metabolites (5), nucleotide metabolism (2), metabolism of cofactors and vitamins (4), metabolism of terpenoids and polyketides (3), translation (1), and signal transduction (1) (Table S8). These results indicate that pathways related to amino acid, carbohydrate, energy, and lipid metabolism are important for Cd responses in leaves of *S. americanum*.

**Figure 6:**
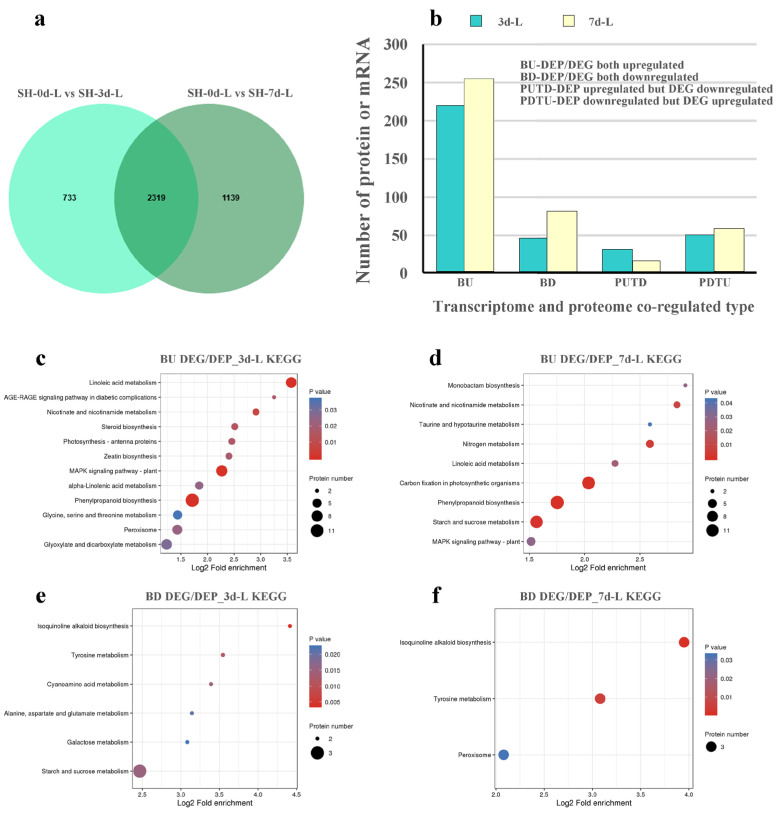
Association analysis of transcriptomic and proteomic data in leaves of *S. americanum* under Cd stress. (**a**) Cd-responsive DEGs/DEPs in leaves at different Cd stress time points; (**b**) the number of co-regulated Cd-responsive DEGs/DEPs; KEGG enrichment analysis of upregulated DEGs/DEPs in leaves under Cd stress for 3 d (**c**) and 7 d (**d**); KEGG enrichment analysis of downregulated DEGs/DEPs in leaves under Cd stress for 3 d (**e**) and 7 d (**f**). “BU” indicates DEP/DEG both upregulated, “BD” indicates DEP/DEG both downregulated, “PUTD” indicates DEP upregulated but DEG downregulated, “PDTU” indicates DEP downregulated but DEG upregulated.

To further explore the Cd response network, an integrated analysis between transcriptomic and proteomic responses was conducted, 2319 proteins/genes were commonly expressed at both different time points (Figure 6a). A total of 144 upregulated and 27 downregulated proteins/genes in leaves showed similar change patterns at transcriptomic and proteomic levels with Cd stress (Figure 6b). However, 22 proteins/genes exhibited opposite change patterns (some proteins were upregulated while their corresponding gene expression were downregulated, or vice versa), including 17 proteins downregulated/genes upregulated, 5 proteins upregulated/genes downregulated (Figure 6b). Go enrichment analysis of proteins/genes exhibited opposite change patterns, revealing that only “iron ion binding” and “transferase activity” were significantly enriched in MF (*p* < 0.05) (Figures S9 and S10). KEGG enrichment analysis found that only “thiamine metabolism” was significantly enriched at both Cd stress time points (*p* < 0.05) (Figure S10). This phenomenon might suggest the involvement of complex post-transcriptional and post-translational regulatory mechanisms, and potential reasons might include differential mRNA stability, translation efficiency, or protein degradation, which may result in a divergence between transcriptomic and proteomic responses of *S. americanum* with Cd stress. Further analysis is needed to explore the specific regulatory processes that govern this discrepancy. At this stage, we focused on proteins/genes with the same change tendency, the GO analysis for upregulated proteins/genes revealed that “oxidoreductase activity” in MF (Figure S7a,b), “chloroplast” in CC (Figure S7c,d), and “green leaf volatile biosynthetic process” in BP (Figure S7e,f) were significantly enriched (*p* < 0.05), while downregulated proteins/genes were significantly (*p* < 0.05) enriched in “single-stranded DNA binding” for MF (Figure S8a,b) and “chloroplast thylakoid lumen” for CC (Figure S8c,d). These results suggest that Cd stress affected oxidoreductase activity, chloroplasts and the photosynthetic process in leaves of *S. americanum*. In addition, KEGG enrichment analysis of proteins/genes with the same trend revealed that common pathways in leaves responding to Cd toxicity at different time points of Cd stress, including “phenylpropanoid biosynthesis”, “linoleic acid metabolism”, “MAPK signaling pathway” and “nicotinate and nicotinamide metabolism” for upregulated proteins/genes (Figure 6c,d), “isoquinoline alkaloid biosynthesis” and “tyrosine metabolism” for downregulated proteins/genes (Figure 6e,f). These findings indicate that the leaves of *S. americanum* can enhance specific biological and metabolic pathways, such as phenylpropanoid biosynthesis, to mitigate Cd stress. Additionally, it can reduce secondary metabolites, such as isoquinoline alkaloid and tyrosine, which are lower-priority, to reduce stress effectively.

**Figure 7:**
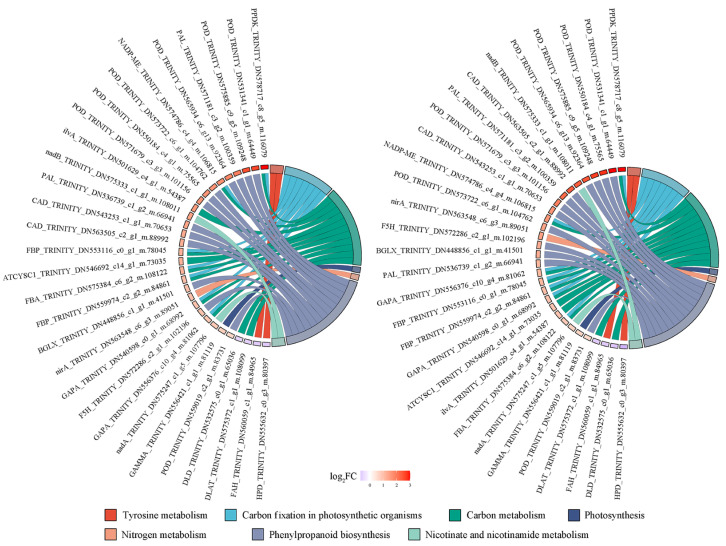
Chord diagrams of DEPs enrichment in leaves of *S. americanum* under Cd stress.

In summary, the joint analysis identified that “phenylpropanoid biosynthesis”, “carbon fixation in photosynthetic organisms”, “carbon metabolism”, “nitrogen metabolism”, “antioxidant activity” and “lipid metabolism” pathways involved in the Cd stress response in leaves of *S. americanum*. Hence, the top 30 significant proteins based on these results were listed (Figure 7), including POD (Peroxidase), PAL (Phenylalanine Ammonia-Lyase), F5H (Ferulate 5-Hydroxylase), CAD (Cinnamyl Alcohol Dehydrogenase), and BGLX (Beta-glucosidase) for phenylpropanoid biosynthesis; PPDK (Pyruvate, orthophosphate dikinase), NADP-ME [Malate dehydrogenase (oxaloacetate-decarboxylating)], GAPA (glyceraldehyde-3-phosphate Dehydrogenase subunit A), FBP (fructose-1,6-bisphosphatase I), and FBA (fructose-bisphosphate aldolase, class I) for carbon fixation in photosynthetic organisms. These DEPs were regarded as key proteins in response to Cd stress in leaves of *S. americanum*. Interestingly, all of them (except for 2 DEPs related to tyrosine metabolism and 2 DEPs related to carbon metabolism) were upregulated.

Moreover, a detailed analysis of phenylpropanoid biosynthesis and carbohydrate metabolism related to photosynthesis was conducted.

**Figure 8:**
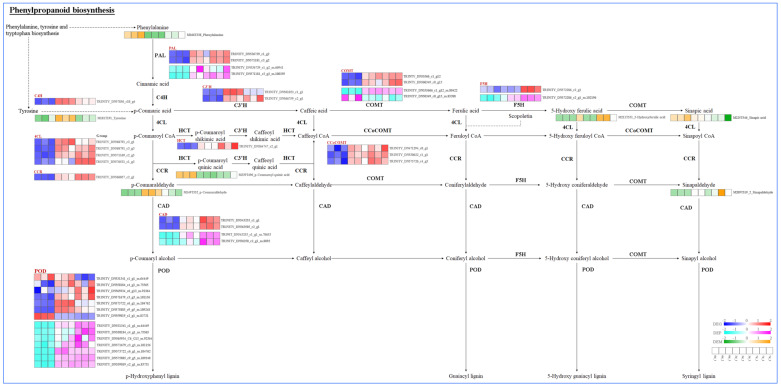
DEGs, DEPs, and DEMs involved in the phenylpropanoid biosynthesis pathway in the leaves of *S. americanum* under Cd stress.

In the Phenylpropanoid biosynthesis pathway (Figure 8), Cd stress increased the expression of genes encoding *PAL*, *CAD*, *POD*, *F5H*, *C4H* (Cinnamate-4-Hydroxylase), *4CL* (4-Coumarate), *CCR* (Cinnamoyl-CoA Reductase), *C3′H* (p-Coumaroyl Shikimate 3-Hydroxylase), *HCT* (Hydroxycinnamoyl-CoA:Shikimate/Quinate Hydroxycinnamoyl Transferase), *COMT* (Catechol-O-methyltransferase), and *CCoCOMT* (Caffeoyl-CoA O-methyltransferase). Additionally, PAL, CAD, POD, COMT, and F5H were upregulated at the protein expression level. Concurrently, tyrosine and p-Coumaraldehyde upregulated at 3 d, 5-Hydroxy ferulic acid and sinapaldehyde upregulated at 7 d, while there was a decreasing trend for phenylalanine, p-Coumaroyl quinic acid, and sinapic acid. Unfortunately, DEMs related to primary monolignols or lignin monomer were not identified. Given the important role of POD in lignin polymerization via the oxidation of monolignols, the upregulation of POD and other rate-limiting enzymes might lead to an increase in lignin content in the leaves of *S. americanum*.

**Figure 9:**
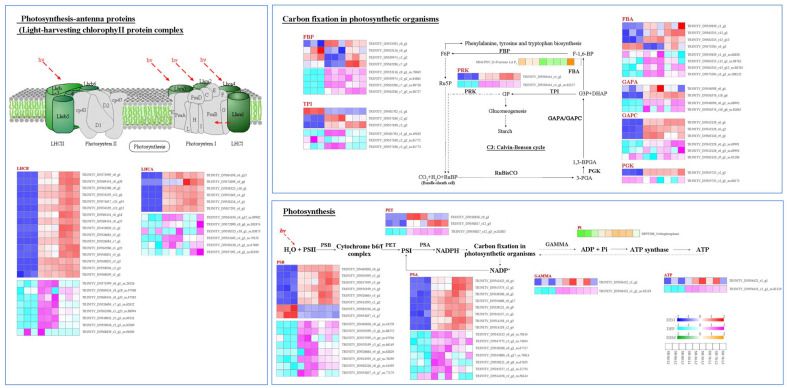
DEGs, DEPs, and DEMs involved in the photosynthesis pathway in the leaves of *S. americanum* under Cd stress.

In carbohydrate metabolism related to photosynthesis, the main processes include photosynthesis, photosynthesis-antenna proteins (light-harvesting chlorophyll II protein complex), and carbon fixation in photosynthetic organisms (Figure 9). For photosynthesis, Cd stress upregulated the gene and protein expression levels of PSB (photosystemII reaction center subunit), PSA (photosystem I reaction center subunit), PET (photosynthetic electron transport), GAMMA (F-type ATPase). DEMs for ATP (adenosine triphosphate) and Pi (orthophosphate) were also upregulated. For photosynthesis-antenna proteins, Cd stress upregulated the gene and protein expression levels of LHC (light-harvesting chlorophyll II protein complex/chlorophyll a-b binding protein), including LHCB and LHCA. For carbon fixation in photosynthetic organisms, gene and protein expression of GAPA, GAPC (glyceraldehyde-3-phosphate dehydrogenase C2), PGK (phosphoglycerate kinase), PRK (phosphoribulokinase) and TPI (triosephosphate isomerase) were upregulated by Cd stress. In addition, gene and protein expression of FBP and FBA were upregulated at 7 d. Concurrently, there was an increasing trend of F-1,6-BP (D-Fructose 1,6-bisphosphate) in response to Cd stress.

**Figure 10:**
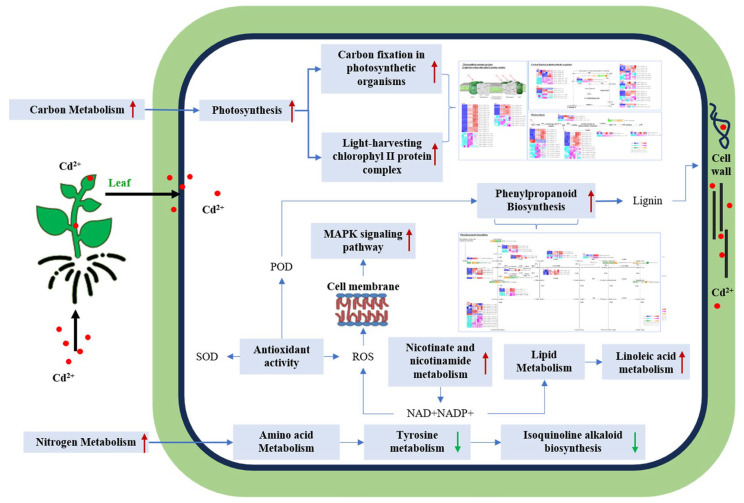
Molecular regulatory network in leaves of *S. americanum* in response to Cd stress. The red dot conceptually represents Cd^2+^.

Based on the multi-omics analyses, a simplified conceptual regulatory model in the leaves of *S. americanum* in response to Cd stress is proposed (Figure 10).

## 3. Discussion

### 3.1. Cd Accumulation Induces Physiological Changes and Enhances Antioxidant Defense in the Leaves of S. americanum

In the current study, seedlings of *S. americanum* were exposed to 160 μM Cd stress, resulting in a reduction in plant biomass. As the duration of Cd stress increased, the Cd concentration increased significantly in the whole plants, including the leaves. Physiological results showed an increase in the content of Chla and Chla/b, while Chlb decreased after 7 d Cd stress. Additionally, SOD activity, POD activity, and MDA content increased concurrently. The accumulation of Cd in the leaves also affected the uptake and distribution of other elements, particularly Mg, Fe, Mn, and K. Cd is a highly toxic HM, which can hinder plant growth and, in severe cases, even lead to death. Cd stress can affect plant growth by altering chloroplasts and inhibiting photosynthesis [13]. Chlorophyll is the foundation for photosynthesis, and Cd stress can hinder its synthesis, as seen in *Sedum alfredii* [16] and *Noccaea caerulescens* [17]. However, for Cd-tolerant species, the photosynthesis process is less affected to some extent [44,45]. Changes in chlorophyll content can indicate the detrimental impacts of stress on photosynthesis [46]. In the present study, the biomass of *S. americanum* decreased under high Cd stress. Woolhouse believes that the Chla/b will decline as leaf age increases [47], but an increasing trend was observed in Chla and Chla/b in the leaves of *S. americanum* (Figure 1c). This might be a result of Cd adaptation, as some plants may increase the relative content of Chla to enhance photosynthetic efficiency under conditions of limited photosynthesis [45,48]. Furthermore, previous studies have indicated that Cd accumulation is closely related to the balance of mineral elements; Cd, a nonessential element, enters plant cell primarily through transporters or metal-chelating proteins for essential metals such as K, Ca, Na, Mn, Zn, and Fe [49,50]. Moreover, Zn, Fe, Cu, Mg, and Mn play vital roles in the process of photosynthesis [49,51]. In our experiment, Mg and Fe content increased, while Mn and Cu content decreased in the leaves of *S. americanum*. These changes in mineral elements may suggest that Cd enters *S. americanum* by competing with the transporters or metal-chelating proteins of Mn and Cu. However, whether these changes directly contribute to the protection of photosynthesis remains speculative and requires further investigation. The observed alterations in mineral content and chlorophyll levels imply that *S. americanum* might cope with Cd toxicity by adjusting ion balance and utilizing specific elements, particularly in relation to photosynthesis, though the exact mechanisms need to be confirmed through future studies.

Cd toxicity can also induce ROS accumulation in plant cells, ROS in plants can serve as signaling molecules [52], and the content of MDA reflects the degree of lipid peroxidation in cell membranes [43,53,54]. Oxygen-scavenging enzymes, such as SOD and POD, can protect plant cells from oxidative damage caused by Cd toxicity [10,55]. The activities of SOD and POD in leaves of *S. americanum* were both increased, consistent with findings in *Abelmoschus manihot* [43] and *Arabis paniculata* [56], indicating that the antioxidant defense system may play important roles in response and tolerance of *S. americanum* to Cd toxicity. The significant increase in MDA content in the leaves indicated that Cd treatment enhances cell membrane lipid peroxidation. Overall, the increased oxygen-scavenging enzyme activity can help *S. americanum* to defense against Cd stress.

### 3.2. S. americanum Copes with Cd Stress by Adjusting Photosynthesis

Based on the integrated analysis of transcriptomes, proteomes, and metabolomes in the leaves, differential responses in pathways related to Cd detoxification were observed, particularly the upregulation in “photosynthesis” under Cd stress.

It has been reported that Cd can influence the photosynthesis process of plants by damaging chloroplast components and affecting the photosynthetic apparatus [57,58]. However, plants have developed multiple defense strategies to respond to Cd stress, and maintaining steady levels of carbon assimilation is a vital solution for enhancing photosynthesis under metal stress [18]. The main components of the light reactions include antenna proteins, PSI, PSII, the cytochrome b6/f complex, and the photosynthetic electron transport chain [30,59]. Antenna proteins are responsible for harvesting light energy and transferring it to the downstream reaction centers. PSII is the core for photosynthesis, responsible for H_2_O oxidation. The PSI and PSII cores are surrounded by LHCS, forming supercomplexes [60,61]. PET links PSII and PSI, with PSI accepting electrons from PSII and using light energy captured by LHCS to drive further electron transfer. This ultimately produces NADPH via the PSA reaction center complex, which provides the key reducing power for the carbon fixation stage of photosynthesis. In the present study, the light-harvesting Chlorophyll protein complex/chlorophyl a-b binding protein increased (Figure 9), as evidenced by the increase in Chla and Chlb content at 3 d (Figure 1). This might indicate that the construction of the photosynthetic reaction center was enhanced under Cd stress. Furthermore, proteins of the PSII reaction center subunit, PSI reaction center subunit, photosynthetic electron transport, and F-type ATPase were also upregulated with Cd stress. This might explain the accumulation of ATP and orthophosphate observed in photosynthesis in this study (Figure 9). Similarly, research has found that 10 μM Cd stress upregulated genes encoding PSBR, PETH, and LHCB in wheat seedlings compared to CK treatment [62], and Cd also upregulated chloroplast genes involved in transcription and translation, such as PSB, PSA, LHCB, and LHCA in the leaves of *Sedum alfredii* [32]. Taken together, the results of our study suggest that short-term Cd stress might enhance photosynthetic efficiency and increase the supply of NADPH and ATP by upregulating the activity of light-harvesting proteins in *S. americanum* leaves.

Carbon fixation in photosynthetic organisms plays a key role in converting CO_2_ into organic matter, which primarily includes carbon fixation, carbon reduction, and ribulose regeneration [63]. The carboxylation process involves CO_2_ combining with Rubisco to produce PGA. The subsequent reduction reaction of PGA further produces Fructose-1,6-bisphosphate (F-1,6-BP) and Fructose-6-phosphate (F6P) through the action of related enzymes, followed by regeneration into Ribose 1,5-diphosphate. We found that F-1,6-BP was upregulated under Cd stress at 7 d. Meanwhile, the expression levels of the DEGs and DEPs of PGK, GAPA, GAPC, FBA, FBP, TPI, and PRK, which regulate the enzymes involved in these reactions, were upregulated (Figure 9). These results suggest that the leaves of *S. americanum* might ameliorate Cd-induced inhibition by upregulating the expression of genes and proteins related to photosynthetic light-harvesting and carbon fixation process.

In summary, photosynthesis plays a vital role in energy metabolism in detoxification against Cd in *S. americanum*.

### 3.3. Phenylpropanoid Biosynthesis Confers Cd Tolerance in the Leaves of S. americanum

Through the integrated analysis of leaves, a notable upregulation of DEGs, DEPs, and DEMs in the “phenylpropanoid biosynthesis” pathway under Cd stress was also identified.

The production of various secondary metabolites, including lignin, flavonoids, tannins, and coumarins, makes phenylpropanoid biosynthesis crucial for Cd resistance [64,65]. Flavonoids, coumarins, and tannins have antioxidant properties that can mitigate oxidative damage caused by Cd-induced ROS accumulation [64]. Lignin, the major component of cell walls, not only strengthens the cell structure but also limits Cd entry into the cytoplasm through ROS-dependent lignification [27]. In this study, combined with metabolome results, Cd increased the levels of tyrosine and p-Coumaraldehyde at 3 d, followed by an increase in 5-Hydroxy ferulic acid and sinapaldehyde at 7 d (Figure 8). p-Coumaraldehyde is an intermediate in lignin biosynthesis, while 5-Hydroxy ferulic acid and sinapaldehyde possess antioxidant activity and may help scavenge ROS induced by Cd. Meanwhile, phenylalanine, p-Coumaroyl quinic acid, and sinapic acid levels decreased. The accumulation of intermediates (such as phenylalanine) is inhibited, while conversion to other downstream metabolites (such as p-Coumaraldehyde, and Sinapaldehyde) occurs. It is hypothesized that *S. americanum* might selectively reduce the synthesis of certain compounds under stress to conserve energy or avoid harmful substance accumulation. While this idea is supported by observed changes in DEMs, it lacks direct empirical evidence for selective metabolism under stress, and further experimental validation is required.

Additionally, key enzymes, such as PAL, C4H, 4CL, CCR, COMT, and POD play essential roles in the synthesis of these metabolites. These enzymes help plants alleviate Cd toxicity directly or indirectly by enhancing plants’ antioxidant capacity and reinforcing the cell wall. PAL, the rate-limiting enzyme, catalyzes the deamination of phenylalanine to produce cinnamic acid, which is the very first step of this pathway. C4H catalyzes the conversion of cinnamic acid to p-coumaric acid. 4CL catalyzes the conversion of p-coumaric acid to 4-coumaroyl-CoA, which is involved in the synthesis of lignin and other secondary metabolites. CCR catalyzes the reduction of cinnamic acid to hydrocinnamic acid, contributing to lignin synthesis. COMT catalyzes the methylation of aromatic compounds such as caffeic acid, leading to the synthesis of lignin precursors and flavonoids, which help plants resist oxidative stress and cellular damage. POD catalyzes the crosslinking of the lignin to form a strong cell wall and plays a role in antioxidant responses. In this study, Cd stress significantly upregulated the gene and protein expression levels of key enzymes, including PAL, CAD, POD, F5H, C4H, 4CL, CCR, C3′H, HCT, COMT, and CCoCOMT (Figure 8), indicating that phenylpropanoid biosynthesis of *S. americanum* is activated in response to Cd stress. Similarly, maize significantly upregulated the gene expression levels of *PAL*, *4CL*, and *CAD* in response to Cd stress [18]. *Abelmoschus manihot* upregulated the gene expression of PAL, C4H, COMT, F5H, 4CL, and POD in response to Cd stress [43]. Exogenous GSH enhances Cd tolerance in *Solanum tuberosum* by upregulating the expression levels of genes and enzymic activities of PAL, CAD, and POD, as well as increasing the lignin content [66]. Additionally, previous studies have shown that the expression levels of the *4CL*, which is involved in phenylpropanoid biosynthesis, are upregulated in response to abiotic stresses [67].

Therefore, we conclude that the phenylpropanoid biosynthesis confers Cd tolerance in the leaves of *S. americanum*.

### 3.4. A Conceptual Response Network in Leaves of S. americanum

Plants, as sessile organisms, must cope with abiotic stress in their growth environment [68]. Cd, one of the most widespread heavy metals, exhibits strong toxicity and typically inhibits plant growth [69,70]. To counteract Cd toxicity, plants, particularly tolerant species or hyperaccumulators, activate various physiological, biochemical, and molecular processes to maintain normal growth [5,56,71].

Our experiment revealed that Cd uptake triggers oxidative stress in leaves (Figure 1), leading to alterations in carbon and nitrogen metabolism (Figure 5 and Figure 10). These changes encompass disruptions in photosynthesis, phenylpropane biosynthesis, induction of oxidative stress, lipid peroxidation in cell membranes, and amino acid metabolisms. Which suggests that the leaves of *S. americanum* might establish a response network by activating signaling pathways, mobilizing antioxidant systems, and adjusting secondary metabolism to maintain physiological homeostasis under Cd stress: antioxidant enzymes, such as SOD and POD, exhibit enhanced activity to scavenge ROS [43,56]; moreover, the upregulation of the MAPK signaling pathway in leaves likely promotes the expression of Cd-stress-responsive genes [72], as this pathway is vital for stress signal transduction [68]; Nicotinate and nicotinamide metabolism, a central pathway for the biosynthesis of NAD^+^ and NADP^+^, is crucial for energy metabolism, redox balance, and stress responses [73,74], the upregulation of this pathway likely supports energy homeostasis with Cd stress [75]; lipid metabolism is critical for defense against Cd stress, potentially through the oxidation of membrane lipids [43,76]. In this study, linoleic acid biosynthesis and steroid hormone biosynthesis pathways were significantly affected by Cd stress. Linoleic acid, a key polyunsaturated fatty acid, contributes to membrane lipids peroxidation in leaves [77], producing intermediate products such as lipid peroxides that serve as signaling molecules in Cd stress responses [78]. Furthermore, products of the steroid hormone biosynthesis such as brassinosteroids (BRs) [79], which regulate cell membrane fluidity and stability, might help leaf cells in mitigating Cd-induced damage [80]. Meanwhile, amino acid metabolism can provide precursors for antioxidant synthesis, such as glutathione (GSH) [81]. In this study, the upregulation of nitrogen metabolism in leaves suggests that plants might respond to Cd stress by upregulated certain amino acid synthesis [82] (such as linoleic acid metabolism). Additionally, products of the taurine and hypotaurine metabolism (such as hypotaurine), which exhibit antioxidant properties [83,84,85], might be involved in Cd-induced reactive oxygen scavenging, although their role has primarily been studied in animals and requires further validation in plants.

Collectively, these physiological and molecular adjustments, particularly the upregulation of photosynthesis and phenylpropanoid pathways mentioned earlier, might enable leaves of *S. americanum* to exhibit robust resilience to Cd stress. These findings elucidate a potential Cd resistance mechanism in the leaves of *S. americanum* and provide a detailed foundation for Cd phytoremediation.

## 4. Materials and Methods

### 4.1. Plant Materials and Hydroponic Treatment

The seeds of *S. americanum* were subjected to surface sterilization by soaking in 0.5% NaClO for 20 min followed by rinsing with deionized water. Subsequently, the water-soaked seeds were transferred into an artificial climate chamber for seedling cultivation, and the seeds were evenly spaced in the 9 cm Petri dishes. Germination occurred under controlled conditions with a temperature setting of 20 °C/15 °C (day/night), a photoperiod of 14/10 h (day/night), a light intensity of 3000 lx, and with the humidity maintained between 55% and 65%. After 2 weeks, when the seedlings had developed 2–4 cotyledons, they were transplanted into an automatic hydroponic culture device using nutrient solution for 30-day adaptive cultivation. Each device contained 3 pots, with 4–6 seedlings per pot, and the biological replicates were 3 to follow physiological parameters determination and multi-omics profiling analysis. The hydroponic culture devices were placed in the greenhouse at Sichuan Agricultural University, Chengdu, China (103°51′ E, 30°42′ N). The temperature of daytime ranged from 29.0 °C to 37.0 °C in the greenhouse, night temperature ranged from 24.5 °C to 28.5 °C, respectively. The relative humidity was 53.0 ± 10.0% throughout the cultivation period. The components of the nutrient solution were as follows: 4 mmol/L Ca(NO3)_2_·4H_2_O, 6 mmol/L KNO_3_, 1 mmol/L NH_4_H_2_PO_4_, 2 mmol/L MgSO_4_·7H_2_O, 80–107 μmol/L Na_2_Fe(EDTA), 46.3 μmol/L H_3_BO_3_, 14.1 μmol/L MnSO_4_, 1.36 μmol/L ZnSO_4_, 0.5 μmol/L CuSO_4_, and 0.01 μmol/L (NH_4_)_6_Mo_7_O_24_. The nutrient solution was renewed every five days during adaptive cultivation.

After adaptive cultivation, *S. americanum* seedlings were subjected to nutrient solution containing CdCl_2_·2.5H_2_O (160 μmol/L). An additional preliminary experiment assessed the photosynthetic physiological parameters of *S. americanum* leaves under a range of Cd concentrations, from 0 to 320 μmol/L CdCl_2_·2.5H_2_O, since it has strong cadmium tolerance, which allowed the level of Cd exposure to be set at 160 μmol/L in the present study [41]. The day of Cd treatment initiation was recorded as day 0, and the Cd stress time lasted 7 days. Samples were collected on day 0 (0 d), day 3 (3 d), and day 7 (7 d) for physiological index measurements and multi-omics analysis. All sample collections were conducted between 8:00 and 11:00 am to minimize the effects of diurnal variation.

### 4.2. Sampling for Physiological Parameters

#### 4.2.1. Biomass and Element Concentration Determination

For physiological parameter samples, the control was plant treated without Cd at the same time points. A total of 3 biological replicates were prepared at each time point for the plant biomass measurement samples, and the harvested plants were washed three times with distilled water. Another 3 biological replicates were prepared at each time point for leaf samples (collected all the leaves of each plant). Plant samples and leaf samples were subsequently oven-dried at 80 °C until constant weight was achieved. The dry weight of each sample was recorded.

The dried samples for element concentration analysis were ground and passed through a 1 mm mesh sieve. Subsamples of approximately 0.2 g were immersed in a mixture of nitric acid (HNO3) and hydrochloric acid (HCl) (*v*:*v*, 4:5) and digested using the microwave digestion system (MARS-5, Thermo Fisher Scientific, Waltham, MA, USA). Subsequently, the concentrations of Cd and other elements (K, Ca, Na, Mg, Mn, Zn, Cu, and Fe) were determined using inductively coupled plasma-mass spectrometry (ICP-MS, Agilent, Santa Clara, CA, USA).

#### 4.2.2. Biochemical Parameters

The fresh leaves (counting the first pair of fully unfold leaves from top to the bottom) from 6 plants were pooled into one sample, and 3 biological replicates were prepared, and the fresh leaf samples were directly used. The malondialdehyde (MDA) content was measured using the TCA-TBA method [83], the activity of superoxide dismutase (SOD) was determined based on the photoreduction of NBT [83], and the activity of peroxidase (POD) was determined using guaiacol substrates, with one unit of enzyme activity expressed as an increase of 0.01 unit of absorbance at 470 nm [84]. The photosynthetic pigments were measured by grinding 0.2 g fresh leaves into a fine powder, which was then suspended in 20 mL of a solution in a mixture of acetone and ethanol (*v*:*v*, 2:1) and then incubated in the dark for 24 h. The concentration of Chla and Chlb contents were determined using a spectrophotometer at 663 and 645 nm [85].

### 4.3. Sampling for Multi-Omics Profiling Analysis

For multi-omics samples, the 0 d sample serves as the control. The fresh leaves (counting the first pair of fully unfold leaves from top to the bottom) from 6 plants were pooled into one sample at each sampling time point, and 3 biological replicates were prepared. All fresh samples were immediately frozen in liquid nitrogen, then stored at −80 °C for further analysis.

#### 4.3.1. Transcriptome Profiling Analysis

(1). RNA extraction and transcriptome sequencing

The transcriptomic analysis was performed using transcriptome sequencing (RNA-seq). A detailed description of RNA extraction, transcriptome sequencing, and data analysis is available in the supporting information Text S1. The expression abundance of all transcripts was determined using RSEM [86] by calculating the fragment per kilobase of transcript per million mapped reads (FPKM). Differential gene expression analysis was performed using Deseq2 [v1.0; https://genepattern.github.io/DESeq2/v1/index.html (accessed on 10 July 2019)]. Genes meeting the following criteria were identified as differentially expressed genes (DEGs): false discovery rate (FDR) ≤ 0.01, and Fold change (FC, Cd-treated/control) ≥ 2 or FC ≤ 0.5.

For DEGs enrichment analysis, GO enrichment analysis of DEGs was implemented by the Goseq R packages [87]. Statistical enrichment of DEGs in KEGG pathways was tested by KOBAS software [v2.0; http://kobas.cbi.pku.edu.cn/ (accessed on 12 July 2019)] [88].

(2). qRT-PCR validation for DEGs

A total of 14 DEGs were randomly selected for qRT-PCR assays to validate the RNA-seq results. Primers were listed in Table S1, and the *18s* and *U6* were selected as housekeeping genes. The PCR system (10 μL) consisted of 1 μL of template cDNA, 0.5 μL of forward primer and 0.5 μL of reverse primer, 5 μL of 2 × SYBR Green Supermix, and 3 μL of ddH_2_O. The reaction program consisted of an initial denaturation step at 95 °C for 3 min followed by 39 cycles of 95 °C for 10 s and 60 °C for 30 s. Melting curves were generated from 60 °C to 95 °C with increments of 1 °C for 4 s. A total of 3 technical replicates were performed for each sample, and transcript levels were calculated using the 2^−ΔΔCt^ method.

#### 4.3.2. Proteome Profiling Analysis

(1). Protein extraction and quantification

The proteomic analysis was performed using Label-free. A detailed description of protein extraction, protein quantification, and data analysis is available in the supporting information Text S2. Proteins meeting the following criteria were considered as differentially expressed proteins (DEPs): unique peptide count ≥ 1, adjusted *p*-value < 0.05, and FC ≥ 1.5 or FC ≤ 0.67.

For DEPs enrichment analysis, GO annotation proteome was derived from the UniProt-GOA database [http://www.ebi.ac.uk/GOA/ (accessed on 25 October 2019)]. GO enrichment analysis for DEPs: Firstly, identified protein ID was converted to UniProt ID, which were then mapped to GO IDs by protein ID. If some identified proteins were not annotated by UniProt-GOA database, the InterProScan software [v.5.14-53.0; http://www.ebi.ac.uk/interpro/ (accessed on 25 October 2019)] would be used to annotate protein’s GO functionality based on the protein sequence alignment method. Then proteins were classified by Gene Ontology annotation based on three categories: biological process, cellular component, and molecular function. For each category, a two-tailed Fisher’s exact test was employed to test the enrichment of DEPs against all identified proteins. The GO with a corrected *p* < 0.05 is considered significant. KEGG database was used to annotate protein pathway. KEGG enrichment analysis of DEPs: Firstly, KEGG online service tools were used to annotate KAAS protein’s KEGG database description. Then, the annotation result was mapped on the KEGG pathway database using KEGG online service tools, such as KEGG mapper. Finally, the KEGG database was used to identify enriched pathways by a two-tailed Fisher’s exact test to test the enrichment of the differentially expressed protein against all identified proteins. The pathway with a corrected *p* < 0.05 was considered significant. These pathways were classified into hierarchical categories according to the KEGG website.

(2). PRM validation for DEPs

A total of 5 DEPs were randomly selected for parallel reaction monitoring (PRM) to validate the LC-MS/MS analysis results. The protein extraction, trypsin digestion, and LC-MS/MS analysis for PRM were performed following the methods described in Text S2.

#### 4.3.3. Metabolome Profiling Analysis

The metabolomic analysis was performed using LC-MS. A detailed description of metabolite extraction, metabolite detection, and data analysis is available in the supporting information Text S3. The Variable importance projection (VIP) was produced by OPLS-DA, and the *p* value was from Student’s t-test. Metabolites meeting the following criteria were considered as differentially expressed metabolites (DEMs): *p* < 0.05 and VIP > 1.

### 4.4. Integrated Analysis of Multi-Omics Data

The transcriptome, proteome, and metabolome data were subjected to statistical analysis to further investigate the relationships between genes, proteins, and metabolites involved in the Cd response. The integrated multi-omics analysis focused on KEGG pathways. Pearson correlation analysis (*p* < 0.01) between DEMs and DEGs or DEPs was performed using tools (http://cloud.keyandaydayup.com/), the selected DEGs, DEPs, and DEMs were then subjected to KEGG analyses. To further investigate the relationship between DEGs and DEPs, an integrated analysis of the transcriptomic and proteomic data was based on the correspondence between mRNA and its translated protein. Finally, the changes in transcripts, proteins, and metabolites were mapped onto KEGG pathways.

### 4.5. Statistical Analysis

Statistical analysis was performed using SPSS 20.0 (SPSS Inc., Chicago, IL, USA). Statistical significance was calculated using one-way ANOVA followed by Tukey’s test and paired sample t-test at the probabilities of *p* < 0.05. Graphs were generated using SigmaPlot 10.0 (Systat Software Inc., San Jose, CA, USA) and Microsoft Excel 2019 (Microsoft, Redmond, WA, USA).

## 5. Conclusions

In this study, it was observed that Cd stress induced physiological changes and enhanced antioxidant defense in the leaves of *S. americanum*. Further analysis of transcriptome, proteome, and metabolome revealed that the upregulation of “photosynthesis” and “phenylpropanoid metabolism” play key roles in coping with Cd toxicity in the leaves of *S. americanum*. Integrative analyses of the transcriptome and proteome identified key proteins responsive to Cd stress, such as POD, PAL, F5H, COMT, GAPA, FBP, and FBA. Subsequently, it was proposed that a molecular regulatory network involving vital genes, proteins, and metabolites respond to Cd toxicity in the leaves. This multi-omics investigation of leaves provides a detailed perspective and molecular database for future research on Cd adaptation or phytoremediation in *S. americanum* and potentially other related plants.

## Data Availability

Data are contained within the article and Supplementary Materials.

## Supplementary Materials

The following supporting information can be downloaded at: https://www.mdpi.com/article/10.3390/plants14142131/s1, Text S1: RNA extraction, transcriptome sequencing, and data analysis; Text S2: Protein extraction, sequencing, and data analysis; Text S3: Metabolites extraction, detection and data analysis; Figure S1: Concentration of K, Ca, Na, Mg, Mn, Zn, Cu, and Fe in leaves of *S. americanum* under Cd stress; Figure S2: Validation of RNA-seq results using qRT-PCR; Figure S3: Validation of label-free results using PRM; Figure S4: GO analysis of upregulated DEPs in leaves of *S. americanum* under Cd stress; Figure S5: GO analysis of downregulated DEPs in leaves of *S. americanum* under Cd stress; Figure S6: PCA and OPLS-DA score plots of all detected metabolites in leaves of *S. americanum* under Cd stress; Figure S7: GO analysis of upregulated proteins/genes in leaves of *S. americanum* under Cd stress; Figure S8: GO analysis of downregulated proteins/genes in leaves of *S. americanum* under Cd stress; Figure S9. GO analysis of downregulated proteins/upregulated genes in leaves of *S. americanum* under Cd stress. Molecular function (a), and biological process (e) enrichment analysis of downregulated proteins/upregulated genes in Cd stress time points at 3d; Molecular function (b), cell component (d), and biological process (f) enrichment analysis of downregulated proteins/upregulated genes in Cd stress time points at 7d. Figure S10. GO analysis of upregulated proteins/downregulated genes in leaves of *S. americanum* under Cd stress. Molecular function (a), cell component (c), and biological process (e) enrichment analysis of upregulated proteins/downregulated genes in Cd stress time points at 3d; Molecular function (b), cell component (d), and biological process (f) enrichment analysis of upregulated proteins/downregulated genes in Cd stress time points at 7d. Figure S11. KEGG enrichment analysis of DEPs/DEGs exhibited opposite change patterns. KEGG enrichment analysis of downregulated proteins/upregulated genes in leaves of *S. americanum* in Cd stress time points at 3d (a) and 7d (b); Table S1: Primer sequences for qPCR analysis of randomly selected DEGs; Table S2: Summary of read statistics from RNA sequencing of *S. americanum*; Table S3: Summary statistics of splicing length; Table S4: Summary statistics of Unigene functional annotations; Table S5: Mass spectrometric data results for *S. americanum*; Table S6: Metabolites classification in leaves of *S. americanum*; Table S7: Common significantly influenced pathways base on metabolites in leaves of *S. americanum* under Cd stress; Table S8: KEGG pathway annotated by Cd-responsive DEG/DEP/DEM in leaves of *S. americanum*.
