# Supplementary material for: Physiological and Multi-Omics Analysis in Leaves of Solanum americanum in Response to Cd Toxicity
Source: Plants (Basel). 2025 Jul 10;14(14):2131. doi: 10.3390/plants14142131 (PMC12299026; doi:10.3390/plants14142131)
Supplement: Supplementary file 1 [file plants-14-02131-s001.zip › Supplementary A-Text.pdf]

Supplementary A

Supplementary A.1

Text S1. RNA extraction, transcriptome sequencing, and data analysis

Total RNA of leaf subsamples was extracted using the Plant RNA Purification Kit (Omega, USA). RNA degradation and contamination were monitored on 1% agarose gels, RNA purity was assessed using the NanoPhotometer spectrophotometer (IMPLEN, CA, USA), RNA concentration was measured using Qubit RNA Assay Kit in Qubit 2.0 Flurometer (Life Technologies, CA, USA), and RNA integrity was assessed using the RNA Nano 6000 Assay Kit of the Agilent Bioanalyzer 2100 system (Agilent Technologies, CA, USA). mRNA was purified from total RNA using poly-T oligo-attached magnetic beads. Fragmentation was carried out using divalent cations under elevated temperature in NEBNext First Strand Synthesis Reaction Buffer (5X). Then, the first-strand cDNA was synthesized using random hexamer primer and M-MuLV Reverse Transcriptase (RNase H), the second-strand cDNA synthesis was subsequently performed using DNA Polymerase I and RNase H. To select cDNA fragments of preferentially 150-200bp in length, the library fragments were purified with AMPure XP system (Beckman Coulter, Beverly, USA). At last, PCR products were purified (AMPure XP system) and library quality was assessed on the Agilent Bioanalyzer 2100 system. The clustering of the index-coded samples was performed on a cBot Cluster Generation System using the TruSeq PE Cluster Kit v3-cBot-HS (Illumia), following the manufacturer's instructions. After cluster generation, the library preparations were sequenced on an Illumina Hiseq™ 2500 platform and paired-end reads were generated.

For quality control, the raw reads of fastq format were firstly processed through in-house perl scripts. In this step, the clean reads were obtained by removing reads containing adapter, reads containing ploy-N and low-quality reads from raw reads. All the downstream analyses were based on clean reads with high-quality. For transcriptome assembly, the high-quality clean reads were subsequently *de novo* assembled using the Trinity package to construct unique consensus sequences as the reference sequence [1]. The assembled unigenes were annotated based on the NCBI non-redundant protein sequences databases (Nr, ftp://ftp.ncbi.nih.gov/blast/db/), Swiss-prot (http://www.uniprot.org/), Kyoto Encyclopedia of Genes and Genomes (KEGG, http://www.genome.jp/kegg/), Gene Ontology (GO, http://geneontology.org/), and Eukaryotic Ortholog Groups (KOG, http://www.ncbi.nlm.nih.gov/KOG/).

Supplementary A.2

Text S2. Protein extraction, sequencing, and data analysis

Proteome quantification based on label-free [2]. For protein extraction, the leaf samples were grinded by liquid nitrogen into cell powder and then transferred to a 5mL centrifuge tube. Then, four volumes of lysis buffer (8mol/L urea, 1% Triton-100, 10mmol/L dithiothreitol, and 1% Protease Inhibitor Cocktail) was added to the cell powder, followed by sonication three times on ice using a high intensity ultrasonic processor (Scientz). The remaining debris was removed by centrifugation at 20,000g at 4°C for 10min. Finally, the protein was precipitated with cold 20% TCA for 2h at -20°C. After centrifugation at 12,000g 4°C for 10min, the supernatant was discarded. The remaining precipitate was washed with cold acetone for three times. The protein was redissolved in 8mol/L urea and the protein concentration was determined with BCA kit according to the manufacturer's instructions. For trypsin digestion, the protein solution was reduced with 5mmol/L dithiothreitol for 30min at 56°C and alkylated with 11mmol/L iodoacetamide for 15min at room temperature in darkness. The protein sample

was then diluted by adding 100mmol/L TEAB to urea concentration less than 2mol/L. Finally, trypsin was added at 1:50 trypsin-to-protein mass ratio for the first digestion overnight and 1:100 trypsin-to-protein mass ratio for a second 4h-digestion.

For LC-MS/MS analysis, the tryptic peptides were dissolved in 0.1% formic acid (solvent A), directly loaded onto a home-made reversed-phase analytical column (15cm length, 75μm i.d.). The gradient was comprised of an increase from 6% to 23% solvent B (0.1% formic acid in 98% acetonitrile) over 26min, 23% to 35% in 8min and climbing to 80% in 3min then holding at 80% for the last 3min, all at a constant flow rate of 400 nl/min on an EASY-nLC 1000 UPLC system. The peptides were subjected to NSI source followed by tandem mass spectrometry (MS/MS) in Q ExactiveTM Plus (Thermo) coupled online to the UPLC. The electrospray voltage applied was 2.0kV. The m/z scan range was 350 to 1800 for full scan, and intact peptides were detected in the Orbitrap at a resolution of 70,000. Peptides were then selected for MS/MS using NCE setting as 28 and the fragments were detected in the Orbitrap at a resolution of 17,500. A data-dependent procedure that alternated between one MS scan followed by 20 MS/MS scans with 15s dynamic exclusion. Automatic gain control (AGC) was set at 5E4. Fixed first mass was set as 100m/z.

For database searching, the resulting MS/MS data were processed using Maxquant search engine (v.1.5.2.8). Tandem mass spectra were searched against human uniprot database concatenated with reverse decoy database. Trypsin/P was specified as cleavage enzyme allowing up to 4 missing cleavages. The mass tolerance for precursor ions was set as 20ppm in First search and 5ppm in Main search, and the mass tolerance for fragment ions was set as 0.02Da. Carbamidomethyl on Cys was specified as fixed modification and acetylation modification and oxidation on Met were specified as variable modifications. FDR was adjusted to <1% and minimum score for modified peptides was set>40.

PRM data analysis for DEPs: For PRM data analysis, the resulting MS data were processed using Skyline (v.20.2). Peptide settings: enzyme was set as Trypsin [KR/P], Max missed cleavage set as 0. The peptide length was set as 7-25, Variable modification was set as Carbamidomethyl on Cys and oxidation on Met. Transition settings: precursor charges were set as 2, 3, ion charges were set as 1, ion types were set as b, y. The product ions were set as from ion 3 to last ion, the ion match tolerance was set as 0.02 Da.

Supplementary A.2

Text S3. Metabolites extraction, detection and data analysis

Metabolite extraction from the frozen leaves samples was conducted following the method established by De et al [3]. Samples were accurately weighed 100mg (±1%) and crushed, after which 0.6mL 2-chlorophenylalanine (4ppm) methanol (-20°C) was added in proportion and thoroughly mixed through homogenization and sonication. Following centrifugation, the supernatant was filtered using a 0.22μm microporous membrane. Subsequently, the supernatant was transferred into the detection bottle for LC-MS detection. The quality control (QC) samples were prepared by combining equal volume (20μL) of all samples and were analyzed in the same manner as the experimental sample.

LC-MS method followed by Zelena et al [4], and want et al [5]. For liquid chromatography conditions, the LC analysis was performed on an Ultimate 3000 UHPLC System (Thermo Fisher Scientific, USA). Chromatography was carried out with an ACQUITY UPLC® HSS T3 (150×2.1mm, 1.8μm) (Waters, Milford, MA, USA). The column maintained at 40°C. The flow rate and injection volume were set at 0.25mL/min and 2μL, respectively. For LC-ESI(+)-MS analysis, the mobile phases consisted of (C) 0.1% formic acid in acetonitrile (v/v) and (D) 0.1% formic acid in water (v/v). Separation was conducted under the following gradient: 0-1min, 2%C; 1-9min, 2%-50%C; 9-12min, 50%-98%C; 12-13.5min, 98%C; 13.5-14min, 98%-2%C; 14-

20min, 2%C. For LC-ESI(-)-MS analysis, the analytes were carried out with (A) acetonitrile and (B) ammonium formate (5mmol/L). Separation was conducted under the following gradient: 0-1min, 2%A; 1-9min, 2%~50%A; 9-12min, 50%-98%A; 12-13.5min, 98%A; 13.5-14min, 98%-2%A; 14-17min, 2%A.

For mass spectrum conditions, mass spectrometric detection of metabolites was performed on Q Exactive (Thermo Fisher Scientific, USA) with ESI ion source. Simultaneous MS1 and MS/MS (Full MS-ddMS2 mode, data-dependent MS/MS) acquisition was used. The parameters were as follows: sheath gas pressure, 30arb; aux gas flow, 10arb; spray voltage, 3.50kV for ESI(+) and -2.50kV for ESI(-), respectively; capillary temperature, 325°C; MS1 range, m/z 100-1000; MS1 resolving power, 70,000 FWHM; number of data dependant scans per cycle, 10; MS/MS resolving power, 17,500 FWHM; normalized collision energy, 30%; dynamic exclusion time, automat.

For data processing and multivariate analysis, the raw data were firstly converted to mzXML format by MSConvert in ProteoWizard software package (v3.0.8789) [6] and processed using XCMS [7] for feature detection, retention time correction and alignment. The metabolites were identified by accuracy mass (<30 ppm) and MS/MS data which were matched with HMDB (http://www.hmdb.ca), massbank (http://www.massbank.jp/), LipidMaps (http://www.lipidmaps.org), mzcloud (https://www.mzcloud.org) and KEGG (http://www.genome.jp/kegg/). The robust LOESS signal correction (QC-RLSC) [8] was applied for data normalization to correct for any systematic bias. After normalization, only ion peaks with relative standard deviations (RSDs) less than 30% in QC were kept to ensure proper metabolite identification.

The Ropls [9] software was used for all multivariate data analyses and modelings. After scaling data, models were built on principal component analysis (PCA), orthogonal partial least-square discriminant analysis (PLS-DA) and partial least-square discriminant analysis (OPLS-DA). The metabolic profiles could be visualized as score plot, where each point represents a sample. The corresponding loading plot and S-plot were generated to provide information on the metabolites that influence clustering of the samples. All the models evaluated were tested for over fitting with methods of permutation tests. The descriptive performance of the models was determined by R2X (cumulative) [perfect model: R2X (cum)=1] and R2Y (cumulative) [perfect model: R2Y (cum)=1] values while their prediction performance was measured by Q2 (cumulative) [perfect model: Q2 (cum)=1] and a permutation test. The permuted model should not be able to predict classes: R2 and Q2 values at the Y-axis intercept must be lower than those of Q2 and the R2 of the non-permuted model.

References

1. Grabherr, M. G.; Haas, B. J.; Yassour, M.; Levin, J. Z.; Thompson, D. A.; Amit, I.; Adiconis, X.; Fan, L.; Raychowdhury, R.; Zeng, Q.; et al. Full-length transcriptome assembly from RNA-Seq data without a reference genome. *Nat. Biotechnol.* **2011**, 29, 644-652.
2. Nanjo, Y.; Nouri, M.; Komatsu, S. Quantitative proteomic analyses of crop seedlings subjected to stress conditions; a commentary. *Phytochemistry* **2011**, 72, 1263-1272.
3. De Vos, R. C.; Moco, S.; Lommen, A.; Keurentjes, J. J.; Bino, R. J.; Hall, R. D. Untargeted large-scale plant metabolomics using liquid chromatography coupled to mass spectrometry. *Nat. Protoc.* **2007**, 2, 778-791.
4. Zelena, E.; Dunn, W.; Broadhurst, D.; Francis-McIntyre, S.; Cain, K.; Begley, P.; O Hagan, S.; Knowles, J.; Halsall, A.; Wilson, I.; et al. Development of a robust and repeatable UPLC-MS method for the long-term metabolomic study of human serum. *Anal. Chem.* **2009**, 81, 1357-1364.

5. Want, E. J.; Masson, P.; Michopoulos, F.; Wilson, I. D.; Theodoridis, G.; Plumb, R. S.; Shockcor, J.; Loftus, N.; Holmes, E.; Nicholson, J. K. Global metabolic profiling of animal and human tissues via UPLC-MS. *Nat. Protoc.* **2013**, 8, 17-32.
6. Smith, C.; Want, E.; O'Maille, G.; Abagyan, R.; Siuzdak, G. XCMS: processing mass spectrometry data for metabolite profiling using nonlinear peak alignment, matching, and identification. *Anal. Chem.* **2006**, 78, 779-787.
7. Navarro, M.; Jaumot, J.; García-Reiriz, A.; Tauler, R. Evaluation of changes induced in rice metabolome by Cd and Cu exposure using LC-MS with XCMS and MCR-ALS data analysis strategies. *Anal. Bioanal. Chem.* **2015**, 407, 8835-8847.
8. Gagnebin, Y.; Tonoli, D.; Lescuyer, P.; Ponte, B.; Seigneux, S.; Martin, P.; Schappler, J.; Boccard, J.; Rudaz, S. Metabolomic analysis of urine samples by UHPLC-QTOF-MS: impact of normalization strategies. *Anal. Chim. Acta* **2016**, 955, 27-35.
9. Thévenot, E. A.; Roux, A.; Xu, Y.; Ezan, E.; Junot, C. Analysis of the human adult urinary metabolome variations with age, body mass index, and gender by implementing a comprehensive workflow for univariate and OPLS statistical analyses. *Journal Proteome Research* **2015**, 14, 3322-3335.
