# Supplementary material for: Physiological and Multi-Omics Analysis in Leaves of Solanum americanum in Response to Cd Toxicity
Source: Plants (Basel). 2025 Jul 10;14(14):2131. doi: 10.3390/plants14142131 (PMC12299026; doi:10.3390/plants14142131)
Supplement: Supplementary file 1 [file plants-14-02131-s001.zip › Supplementary B-Figure add S9-S11.pdf]

Supplementary B

Supplementary B.1

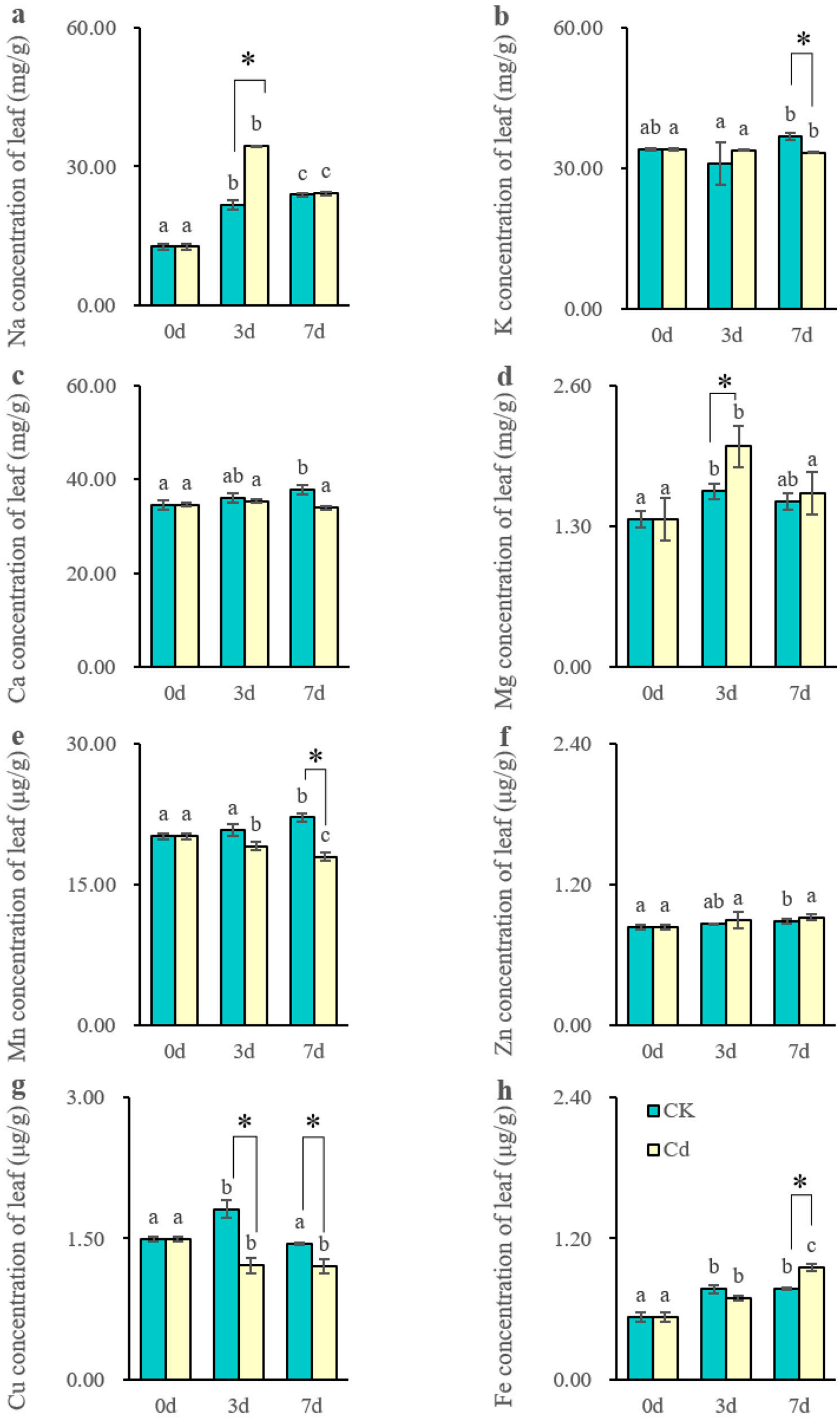

Figure S1. Concentration of K, Ca, Na, Mg, Mn, Zn, Cu, and Fe in leaves of *S. americanum* under Cd stress.

Supplementary B.2

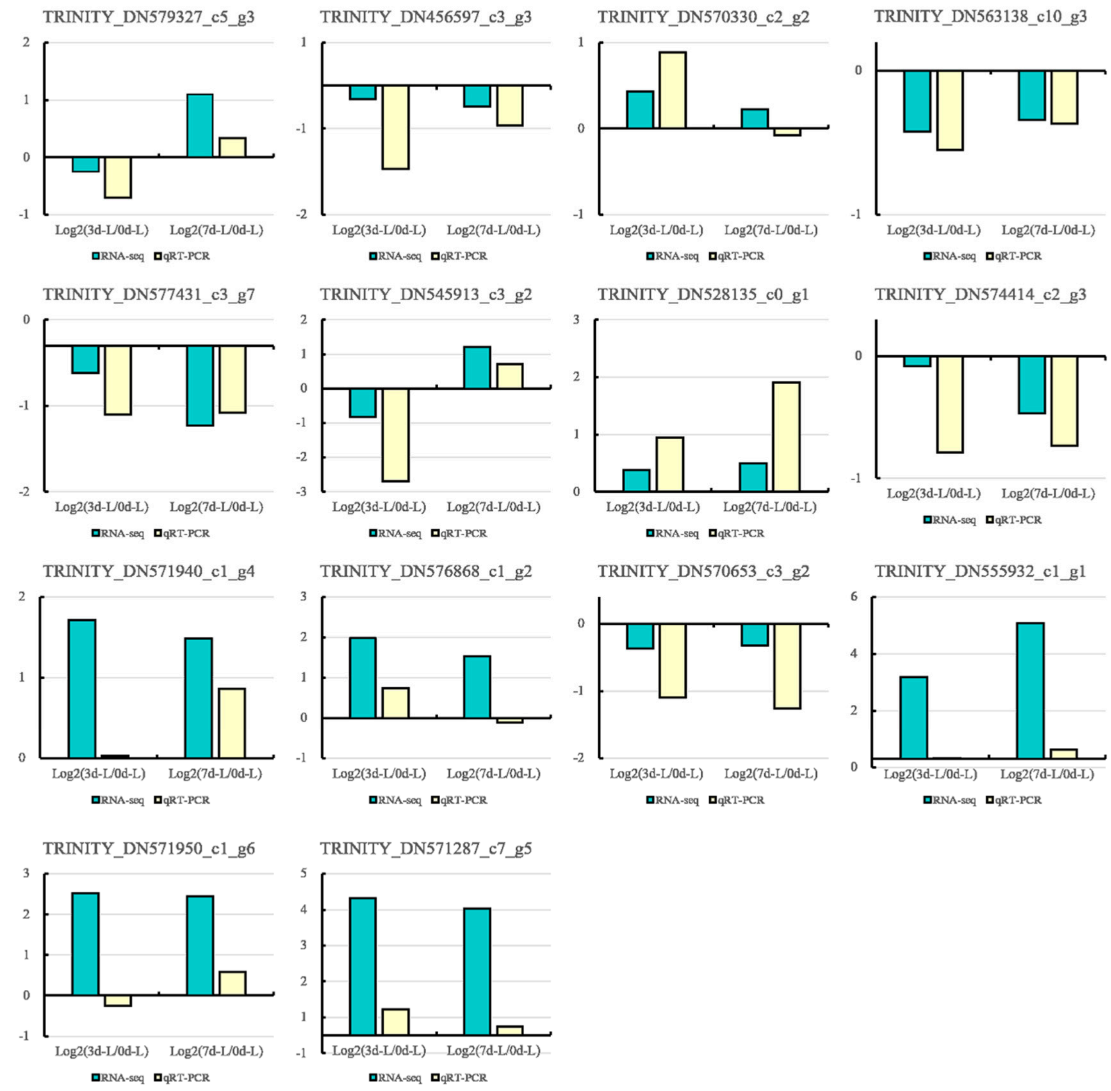

Figure S2. Validation of RNA-seq results using qRT-PCR.

Supplementary B.3

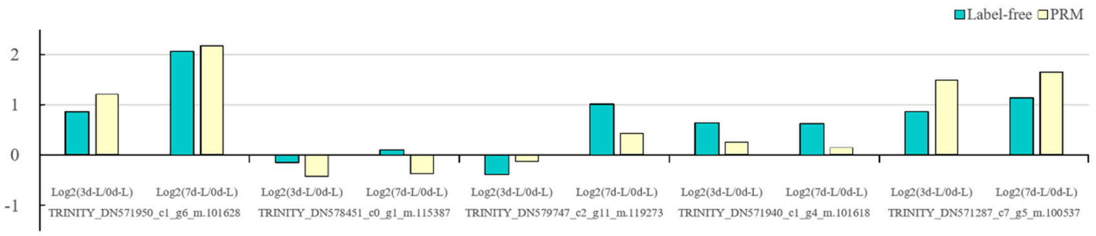

Figure S3. Validation of label-free results using PRM.

Supplementary B.4

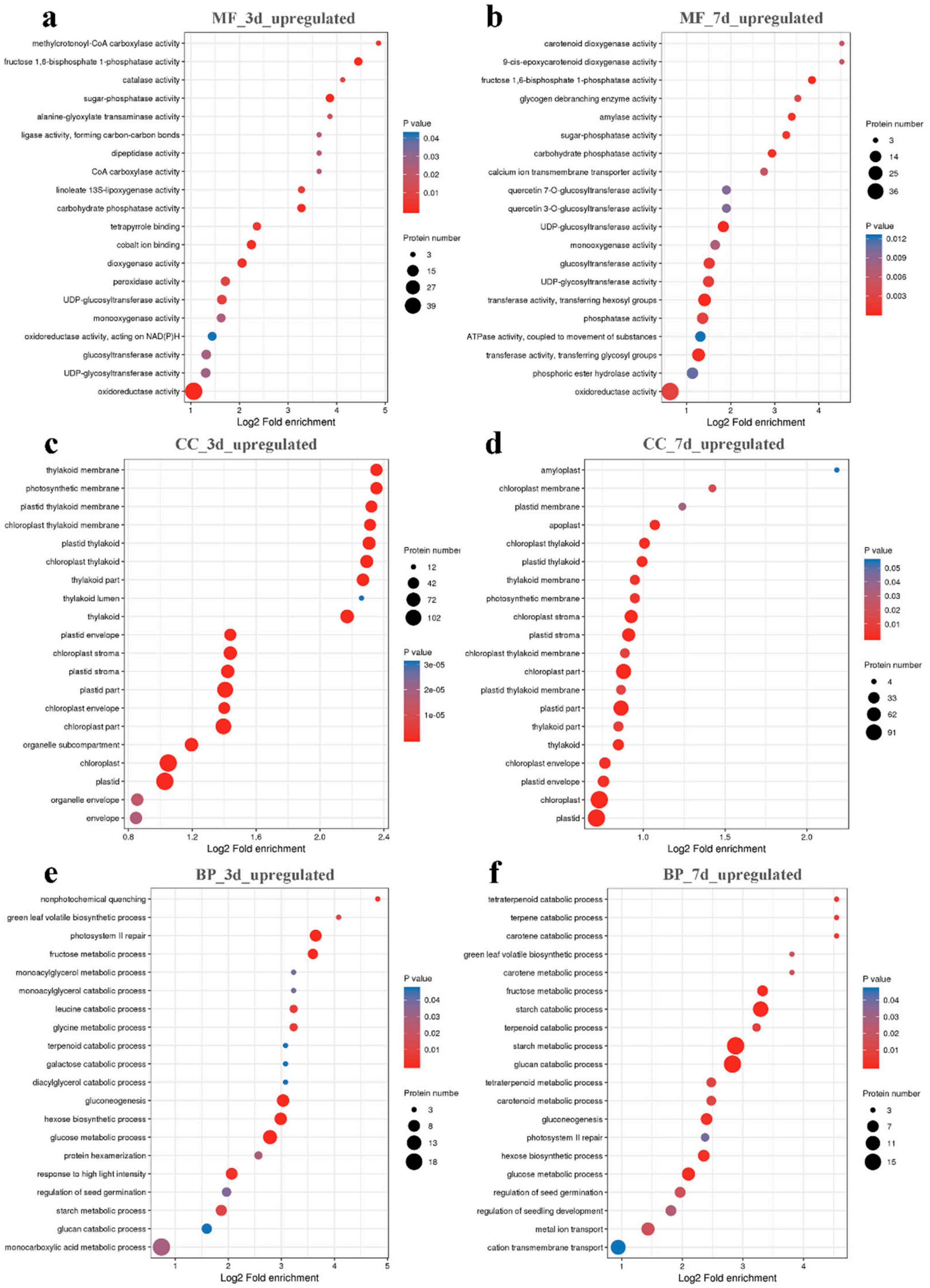

Figure S4. GO analysis of upregulated DEPs in leaves of *S. americanum* under Cd stress. GO enrichment of upregulated DEPs in molecular function category at 3d (a) and 7d (b); GO enrichment of upregulated DEPs in cellular component category at 3d (c) and 7d (d); GO enrichment of upregulated DEPs in biological process category at 3d (e) and 7d (f).

Supplementary B.5

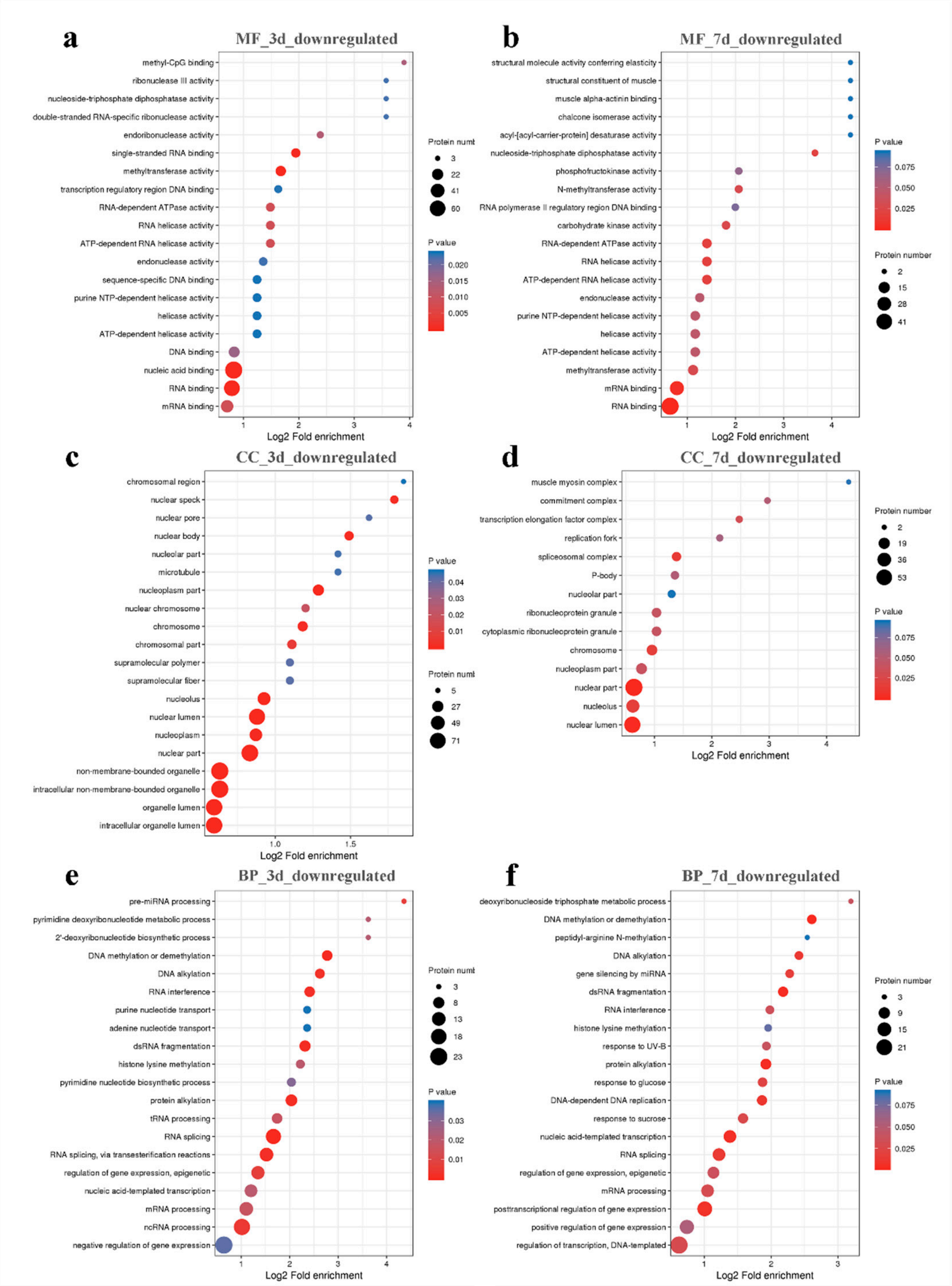

Figure S5. GO analysis of downregulated DEPs in leaves of *S. americanum* under Cd stress. GO enrichment of downregulated DEPs in molecular function category at 3d (a) and 7d (b); GO enrichment of downregulated DEPs in the cellular component category of 3d (c) and 7d (d); GO enrichment of downregulated DEPs in biological process category at 3d (e) and 7d (f).

Supplementary B.6

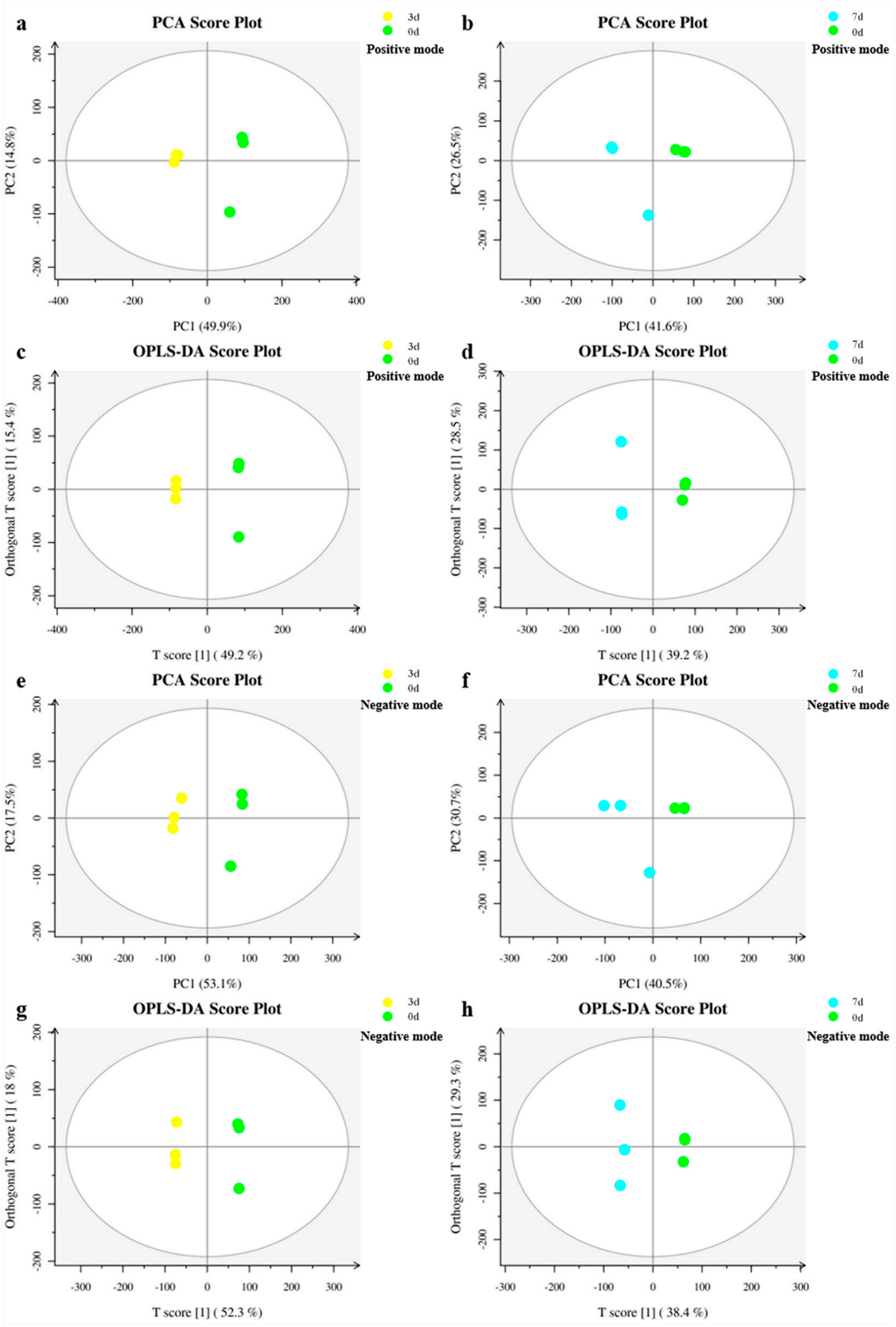

Figure S6. PCA and OPLS-DA score plots of all detected metabolites in leaves of *S. americanum* under Cd stress. PCA (a,b) and OPLS-DA (c,d) score plots of all detected metabolites in different Cd treatment groups (160 μmol/L Cd stress time points of 0d, 3d and 7d), with data shown in a positive (ESI+) ion mode; PCA (e,f) and OPLS-DA (g-h) score plots of all detected metabolites in different Cd treatment groups (160 μmol/L Cd stress time points of 0d, 3d and 7d), with data shown in a negative (ESI-) ion mode.

Supplementary B.7

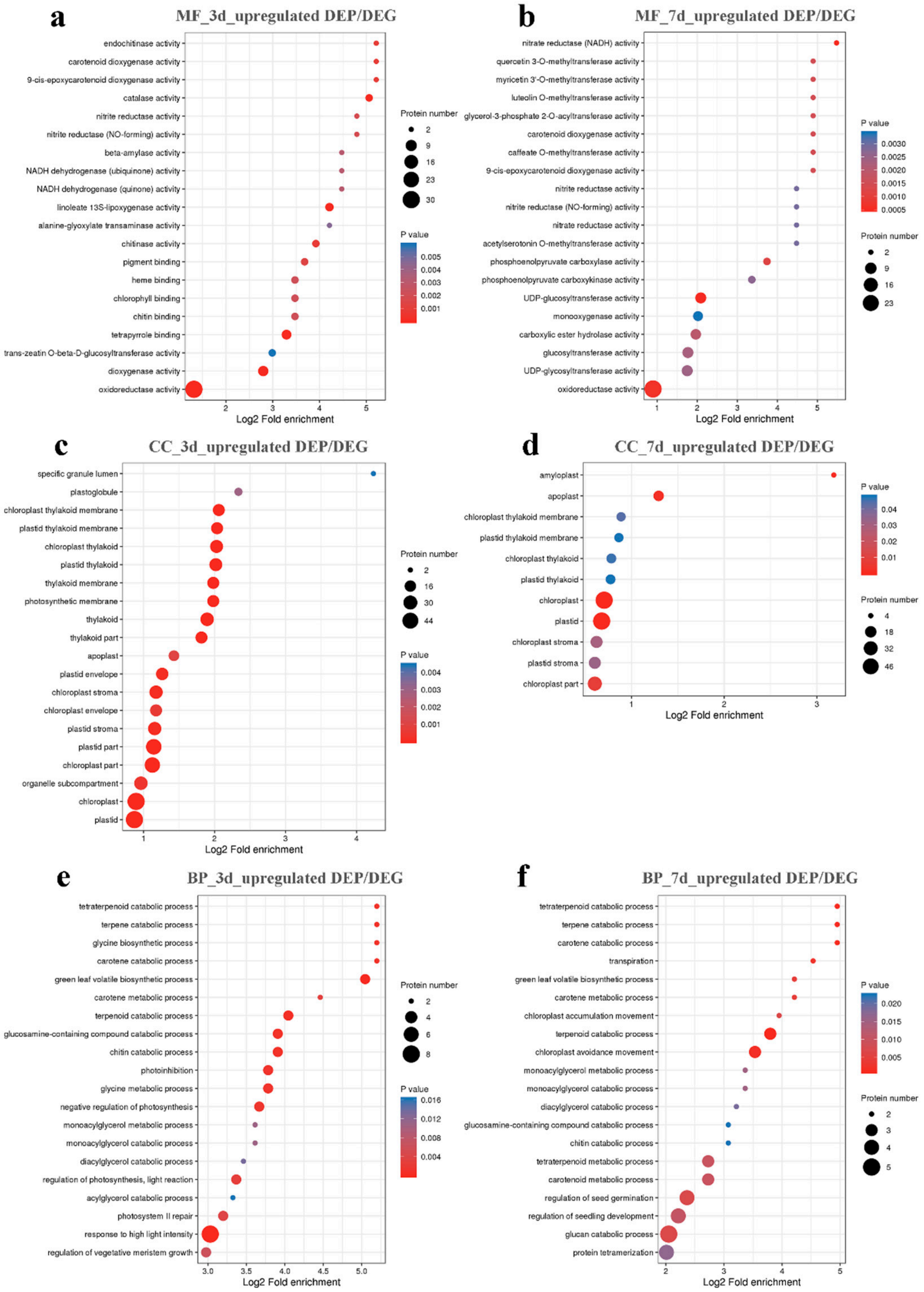

Figure S7. GO analysis of upregulated proteins/genes in leaves of *S. americanum* under Cd stress. Molecular function (a), cell component (c), and biological process (e) enrichment analysis of upregulated proteins/genes in Cd stress time points at 3d; Molecular function (b), cell component (d), and bio-logical process (f) enrichment analysis of upregulated proteins/genes in Cd stress time points at 7d.

Supplementary B.8

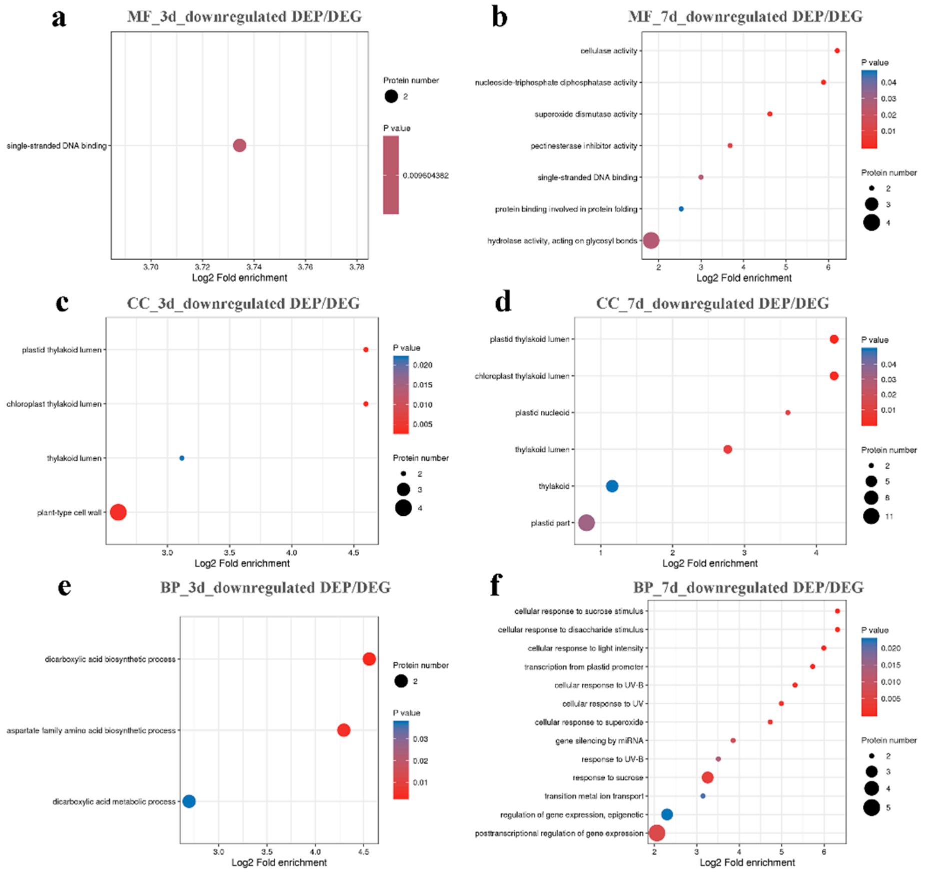

Figure S8. GO analysis of downregulated proteins/genes in leaves of *S. americanum* under Cd stress. Molecular function (a), cell component (c), and biological process (e) enrichment analysis of downregulated proteins/genes in Cd stress time points at 3d; Molecular function (b), cell component (d), and bio-logical process (f) enrichment analysis of downregulated proteins/genes in Cd stress time points at 7d.

Supplementary B.9

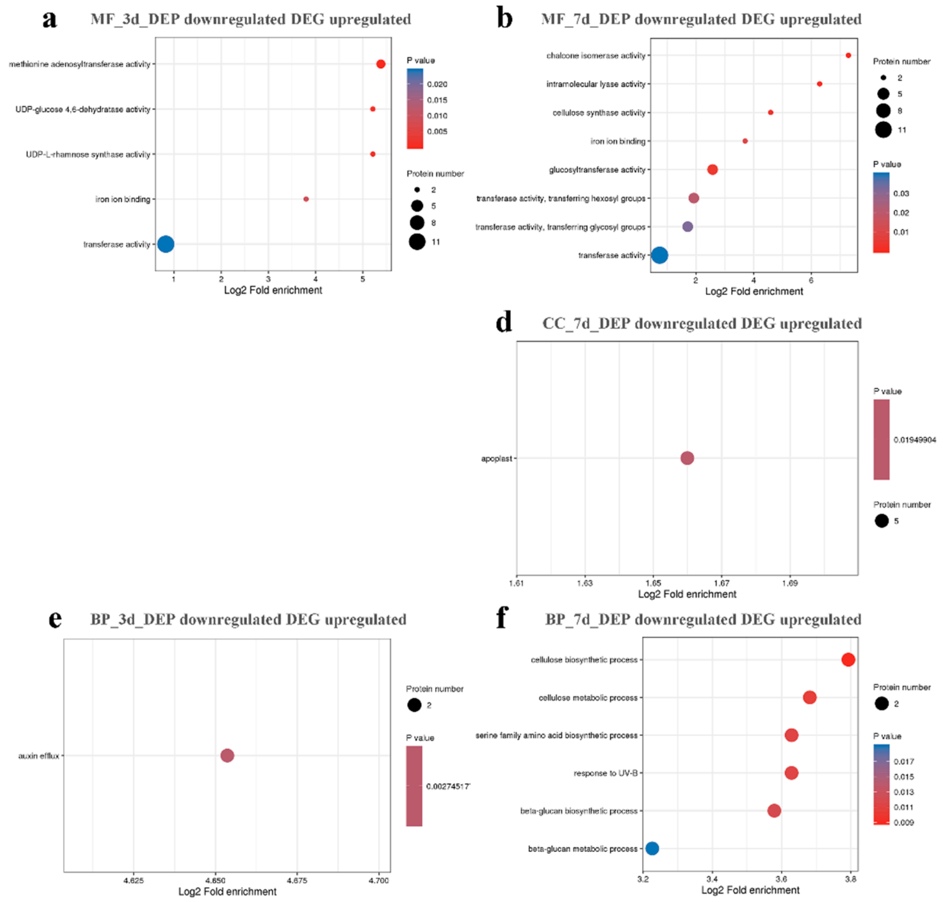

Figure S9. GO analysis of downregulated proteins/upregulated genes in leaves of *S. americanum* under Cd stress. Molecular function (a), and biological process (e) enrichment analysis of downregulated proteins/upregulated genes in Cd stress time points at 3d; Molecular function (b), cell component (d), and biological process (f) enrichment analysis of downregulated proteins/upregulated genes in Cd stress time points at 7d.

Supplementary B.10

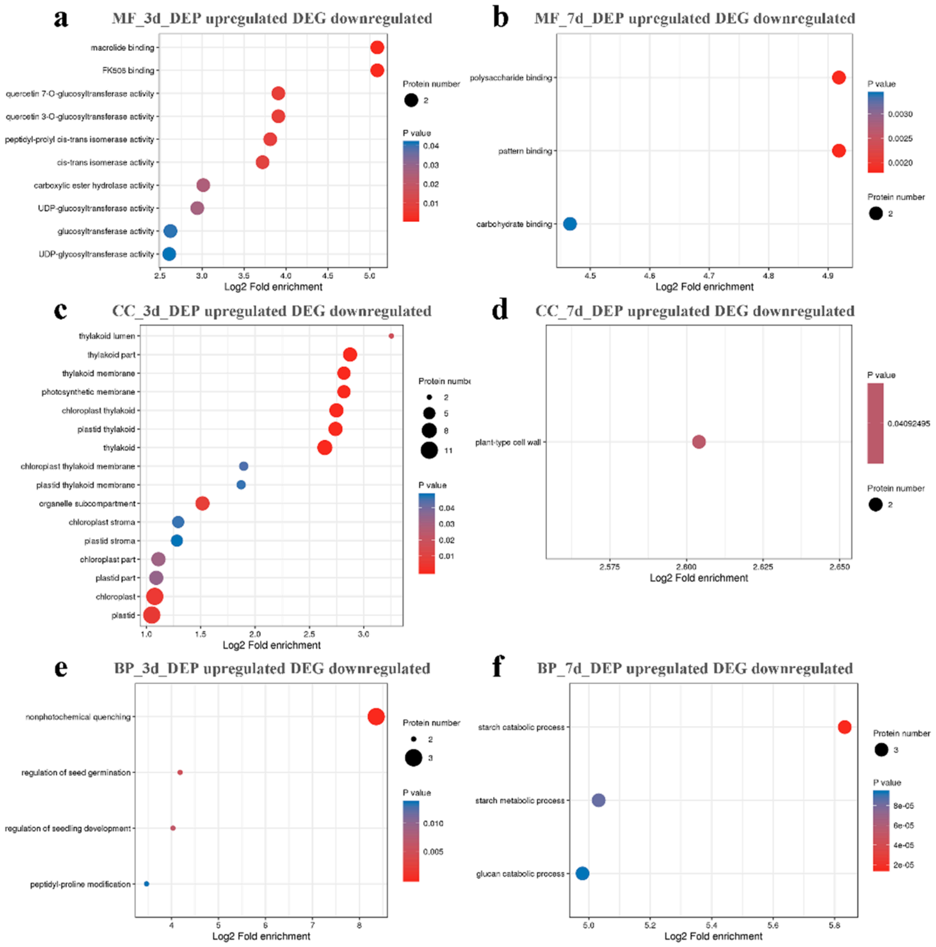

Figure S10. GO analysis of upregulated proteins/downregulated genes in leaves of *S. americanum* under Cd stress. Molecular function (a), cell component (c), and biological process (e) enrichment analysis of upregulated proteins/downregulated genes in Cd stress time points at 3d; Molecular function (b), cell component (d), and biological process (f) enrichment analysis of upregulated proteins/downregulated genes in Cd stress time points at 7d.

Supplementary B.11

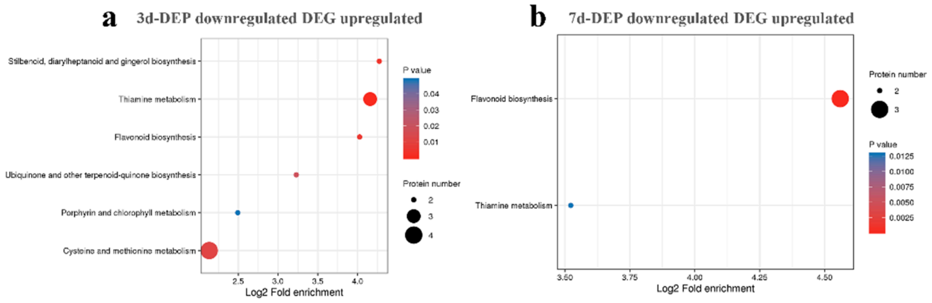

Figure S11. KEGG enrichment analysis of DEPs/DEGs exhibited opposite change patterns. KEGG enrichment analysis of downregulated proteins/upregulated genes in leaves of *S. americanum* in Cd stress time points at 3d (a) and 7d (b).
